# Microenvironmental Effects on CO_2_ Hydrogenation Over PdZn Alloy Catalysts

**DOI:** 10.1002/advs.202509726

**Published:** 2025-09-14

**Authors:** Jikai Sun, Jianzhong Wu

**Affiliations:** ^1^ Department of Chemical and Environmental Engineering University of California Riverside CA 92521 USA

**Keywords:** classical density function theory, CO_2_ hydrogenation, microenvironmental effect

## Abstract

Understanding catalytic reactions under realistic gas‐phase conditions is essential for the computational design of next‐generation industrial catalysts with enhanced efficiency and selectivity. In this work, a hybrid quantum/classical framework is employed to systematically investigate the effects of partial pressure, temperature, and surface coverage on CO_2_ hydrogenation over PdZn alloy catalysts. The multiscale approach incorporates local gas‐phase densities and realistic catalyst structures, enabling accurate prediction of reaction selectivity across operating conditions. The results reveal a temperature‐dependent shift in pressure response: at low temperatures, increasing pressure favors the COOH pathway, while at elevated temperatures, the HCOO pathway becomes more competitive as pressure increases. This opposite trend reflects a competition of long‐range and short‐range interactions between gas molecules and intermediates, which evolves nonlinearly with the system pressure. The surface structure further modulates catalyst‐environment interactions by altering local gas accessibility, for example, through suppressing CO_2_ adsorption while promoting the catalyst binding of smaller molecules like H_2_. These findings provide a detailed mechanistic understanding of how catalyst structure and reaction environment jointly regulate free‐energy pathways in CO_2_ hydrogenation. The transferable strategy of integrating microenvironmental effects into first‐principles modeling advances the rational design of catalytic systems for efficient CO_2_ utilization and broader chemical transformations.

## Graphical abstract

Interactions between intermediates, adsorbates, and gas‐phase molecules dictate the selectivity of CO_2_ hydrogenation.

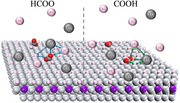

## 1 Introduction

Thermocatalytic hydrogenation of CO_2_ into high‐value chemicals holds significant promise for carbon emission reduction and sustainable development.^[^ ^1^, ^2^, ^3^, ^4^ ^]^ However, the reaction network of CO_2_ hydrogenation is inherently complex, depending on a wide range of factors‐including catalyst structure, temperature, pressure, and gas‐phase composition. Even for a relatively simple product like formic acid (HCOOH), there are three primary reaction pathways, each evolving the addition of two hydrogen atoms.^[^ ^5^ ^]^ As more hydrogen atoms are added, the reaction network becomes increasingly complex, making CO_2_ hydrogenation difficult to decipher due to the diverse range of products.^[^ ^6^, ^7^, ^8^ ^]^

Understanding reaction mechanisms under realistic gas‐phase conditions is essential for the rational design of industrial catalysts with enhanced activity and tunable selectivity.^[^ ^9^, ^10^ ^]^ Experimentally, identifying reaction intermediates through various characterization techniques offers valuable insights into catalytic processes.^[^ ^11^, ^12^ ^]^ However, the harsh reaction conditions and the transient nature of unstable intermediates make it challenging to determine reaction pathways experimentally.^[^ ^13^, ^14^, ^15^ ^]^ In recent years, quantum‐chemistry calculations have made significant progress in describing reaction mechanisms, enabling the theoretical modeling of industrial catalysts and systematic exploration of reaction mechanisms.^[^ ^8^, ^16^, ^17^, ^18^, ^19^, ^20^ ^]^ The first‐principles approach is mostly based on the Kohn–Sham density functional theory (KS‐DFT), with various approximations for the exchange‐correlation energy.^[^ ^21^ ^]^ Due to the high computational cost in the calculation of electronic density, the theoretical model is typically limited to the nanometer scale.^[^ ^5^ ^]^ As a result, KS‐DFT simulations of heterogeneous gas–solid reactions are often performed in vacuum, neglecting the pressure and compositional effects that influence interactions between adsorbates and surrounding gas‐phase molecules. As the intermolecular and surface interactions are affected by the local gas density, microenvironmental effects become increasingly significant under high‐pressure conditions.^[^ ^22^, ^23^ ^]^

The limitations of first‐principles methods to describe reaction environments in heterogeneous catalysis lead to two well‐known discrepancies between theory and experiment, commonly referred to as the “materials gap” and the “pressure gap”.^[^ ^24^ ^]^ These gaps are largely responsible for significant errors in theoretical predictions of catalytic activity and selectivity. The materials gap stems from challenges in identifying active sites and capturing the dynamic structure of catalysts in real‐world applications.^[^ ^25^, ^26^, ^27^, ^28^, ^29^, ^30^ ^]^ As noted by Schwarzer and coworkers,^[^ ^31^ ^]^ heterogeneous catalysts often develop active structures under reaction conditions involving high temperatures and pressures, making them inaccessible to observation using low‐temperature, ultra‐high‐vacuum techniques. To address this issue, a growing number of studies have been focusing on the dynamic evolution of catalysts under realistic reaction conditions.^[^ ^28^, ^32^ ^]^ An illustrative example was reported by Wang et al.,^[^ ^33^ ^]^ concerning CO oxidation catalyzed by Au clusters. The Au catalyst undergoes dynamic structural changes, with Au⁺ ions detaching from the nanoparticles to catalyze the oxidation reaction at the metal/oxide interface, and subsequently reintegrating into the nanoparticles once the reaction is complete.^[^ ^33^ ^]^ Recently, Zhang and Liu reported the surface phase diagrams of PdZn alloys for CO_2_ hydrogenation under a wide range of temperatures and pressures.^[^ ^34^, ^35^ ^]^ It was found that the surface coverage varies significantly with the reaction environment, which in turn leads to different catalytic activities and selectivity. However, even after accounting for surface coverage by various species, the *COOH pathway was identified as the dominant route for both CO and HCOOH (CH_3_OH) formation on PdZn. This finding contrasts with numerous experimental studies that emphasize the prominent role of the formate pathway in CO_2_ hydrogenation, which is recognized for its critical influence on product selectivity on PdZn alloys.^[^ ^36^, ^37^, ^38^, ^39^ ^]^ To account for the pressure effect, conventional first‐principles methods typically incorporate the gas‐phase pressure by applying an ideal‐gas correction to the reaction free energy. The ideal‐gas approach neglects both interactions between gas‐phase species and their inhomogeneous distribution near the catalyst surface.^[^ ^22^, ^40^ ^]^

A faithful representation of industrial catalytic systems requires accurate modeling of both interfacial structure and chemical reactivity. While the former depends on catalyst‐environment interactions and varies with thermodynamic conditions, the latter is dictated by the electronic properties of reactive species underlying surface reactions. In a previous work, we proposed a hybrid method that combines the classical density functional theory (cDFT) to capture the thermodynamic effects with the Kohn‐Sham (KS) DFT for predicting catalyst structure and reactivity.^[^ ^41^ ^]^ Compared with conventional first‐principles methods, a key distinction of the multiscale approach lies in the thermodynamic ensemble employed: cDFT calculations are performed within the grand canonical (µVT) ensemble, enabling the direct simulation of the reaction environment. This hybrid framework allows for a more realistic treatment of gas‐phase species, especially under varying pressures and chemical compositions. Leveraging this theoretical capability, we have investigated CO_2_ hydrogenation to methanol at Cu surfaces over a broad range of thermodynamic conditions.^[^ ^41^ ^]^ Our results reveal that, due to the strong attraction of gas‐phase species to the catalyst surface, the local gas density can reach several hundred times its bulk value. This surface enrichment leads to non‐negligible surface energies—on the order of several kcal/mol—that must be considered for accurate modeling of reaction mechanisms. Conventional models, which often neglect microenvironmental contributions, may fail to capture key aspects of catalytic behavior under realistic conditions.

Building on our previous work, this study aims to address the coupling effects of catalyst structure and reaction environment on CO_2_ hydrogenation. By explicitly considering the influence of surface coverage on the electronic structure, the extended theoretical framework enables us to account for complex interactions among surface‐bound species, reaction intermediates, and gas‐phase molecules that are neglected by conventional KS‐DFT studies. To showcase these new capabilities, we use CO_2_ hydrogenation over PdZn alloy catalysts as a prototype system and investigate the effect of surface morphology on the catalytic behavior under realistic experimental conditions.

To highlight the key novelty of this work, we analyzed the temperature‐dependent relationship between reaction selectivity and gas‐phase pressure, as well as how this relationship evolves with varying surface coverages. We defined a suite of methodologies—including gas‐phase surface density maps, differential density analysis for configurational transitions and environmental evolution, and visualization techniques—to elucidate the underlying theoretical mechanisms governing interactions between gas‐phase molecules and surface‐bound intermediates. Our approach distinguishes between short‐range and long‐range interactions between gas‐phase species and surface adsorption configurations. Furthermore, we explored how the local gas‐phase composition dynamically responds to changes in surface intermediate configurations and external reaction conditions. This investigation allowed us to uncover general trends governing the evolution of gas–intermediate interactions under realistic catalytic environments.

## 2 Results

**Figure 1:**
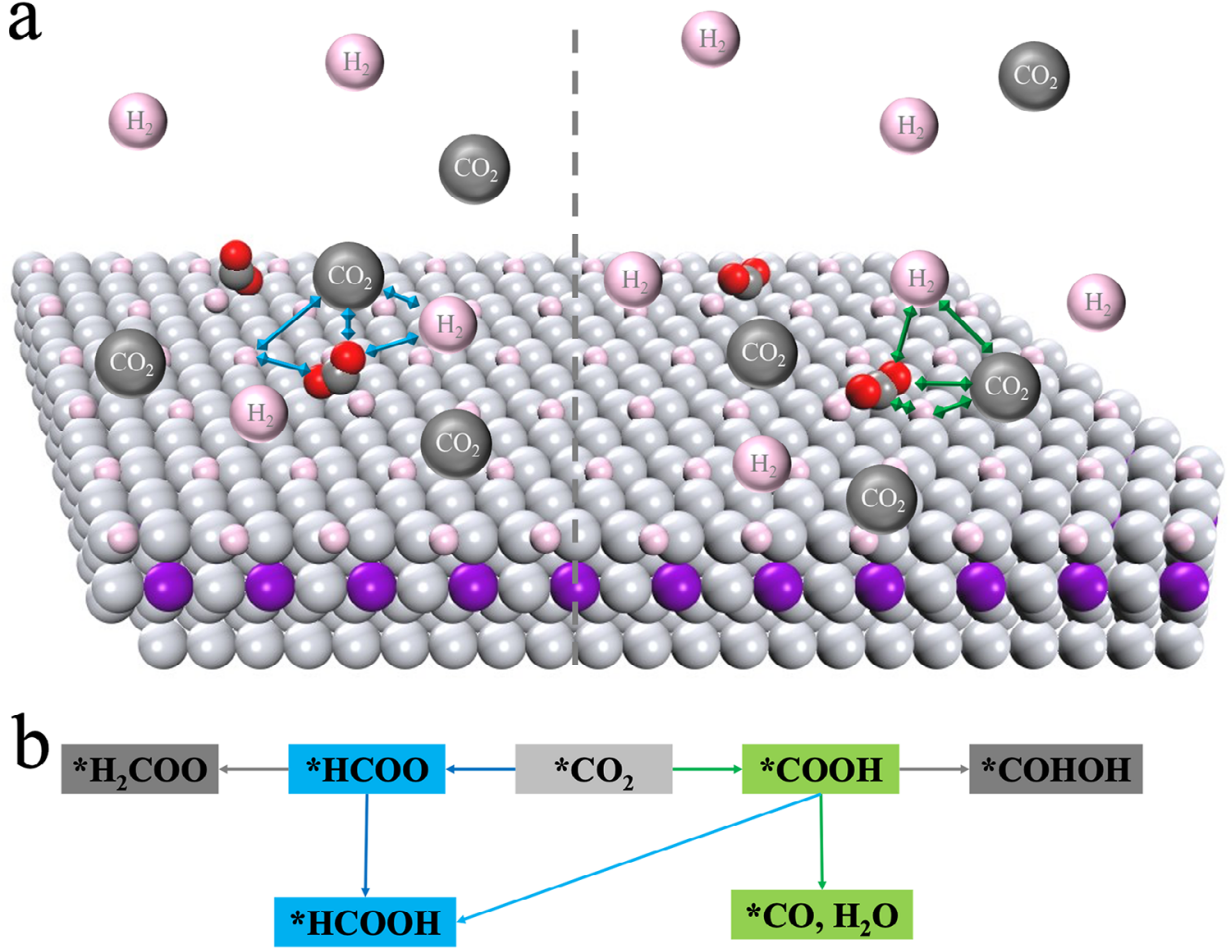
a) Schematic illustration of surface intermediates and microenvironment for CO_2_ hydrogenation over PdZn alloy catalysts. The grey and purple balls are Pd and Zn atoms, respectively. The pink balls are dissociated H atoms. b) Possible reaction pathways involving the addition of two hydrogen atoms to a CO_2_ molecule.

In this work, we used a hybrid DFT framework to systematically investigate the effects of temperature, pressure, and gas‐phase composition on the selectivity of dihydrogenated products from CO_2_ hydrogenation over PdZn alloy surfaces with varying surface coverages. **Figure** **1**a illustrates an embedding scheme that facilitates the combination of KS‐DFT and cDFT calculations. The first‐principles method was used to determine the catalyst surface structure by identifying the atomic configuration of all adsorbates and active sites through energy minimization in vacuum. Meanwhile, cDFT was used to account for the effects of reaction environment within a grand canonical ensemble defined by temperature, the chemical potentials of all gas species, and the total volume of the reaction system.

Throughout this work, the system volume is defined by a simulation box representing the catalyst in contact with CO_2_ and H_2_ at different temperatures, pressures, and bulk compositions. The gas phase is modeled as an open space extending 5 nm in the direction perpendicular to the catalyst surface. The atomic structures and electronic energies of all intermediates were adopted from the KS‐DFT calculations conducted by Zhang and Liu.^[^ ^34^, ^35^ ^]^ Building upon their theoretical investigations into the dynamic structures of PdZn catalysts, we examined how the catalyst structures interact with environmental conditions, influencing the catalyst performance and reaction pathways. Because the surface structure and gas‐phase molecular distributions are decoupled in the hybrid DFT calculations, we can determine independently the electronic energies of various surface‐bound intermediates and their interactions with gas‐phase species at different thermodynamic conditions.

Figure 1b presents possible reaction pathways for CO_2_ hydrogenation involving the addition of two hydrogen atoms to a CO_2_ molecule.^[^ ^5^, ^15^, ^39^ ^]^ Three distinct routes have been identified, even without considering other dihydrogenated intermediates, leading to the production of CO+H_2_O and HCOOH, respectively. Moreover, the overall reaction selectivity is sensitive to whether CO and HCOOH desorb from the surface as final products or undergo further hydrogenation.

### 2.1 Non‐Monomeric Temperature Effect on CO_2_ Hydrogenation

**Figure 2:**
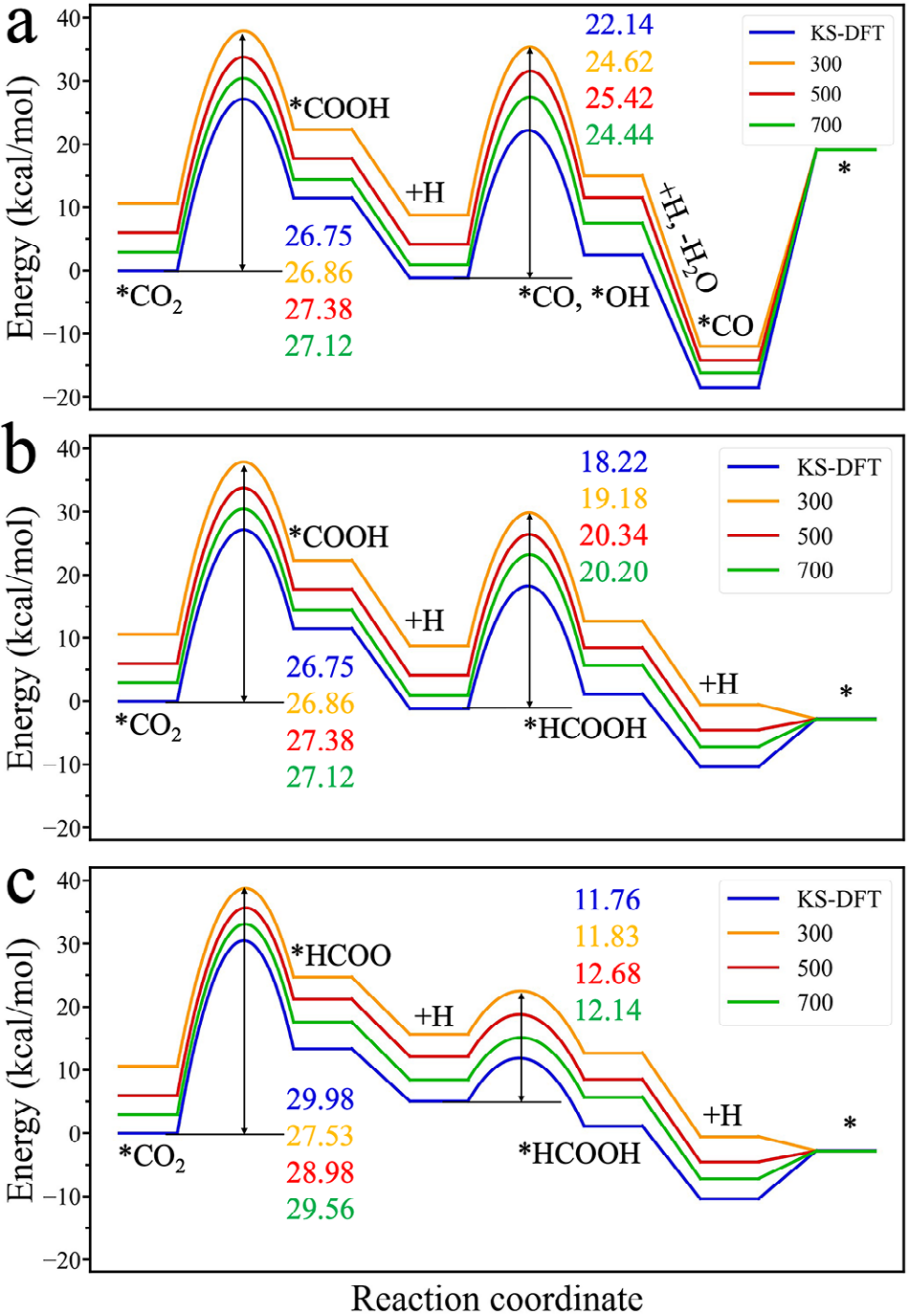
The (free) energy profiles for CO_2_ hydrogenation over PdZn_H025. a) CO formation via CO_2_ hydrogenation. b) HCOOH formation via the *COOH pathway. c) HCOOH formation via the *HCOO pathway. The blacklines are the ground‐state energies at 0 K predicted by KS‐DFT, the yellow, red, and green lines are the grand potentials (Ω) calculated from the hybrid DFT under the conditions of bulk gas‐phase partial pressures P_CO2_ = 10 bar and P_H2_ = 30 bar at temperatures of 300, 500, and 700 K, respectively.

We first consider the temperature effects on the free‐energy landscapes of three representative reaction pathways for CO_2_ hydrogenation, leading to the production of CO+H_2_O and HCOOH. **Figure** **2** summarizes the key results from KS‐DFT and hybrid DFT calculations for a Zn‐modified Pd(111) surface (PdZn_H025, following the notation of Zhang and Liu.^[^ ^34^, ^35^ ^]^), which features 0.25 coverage of adsorbed hydrogen (H*). Here, the KS‐DFT results correspond to the reaction at 0 K, while the cDFT calculations were conducted under gas‐phase conditions relevant to industrial CO_2_ hydrogenation processes, with bulk partial pressures of P_H2_ = 30 bar and P_CO2_ = 10 bar and temperatures at 300, 500, and 700 K. To relate the energy landscape to reaction selectivity, our discussions are focused primarily on the rate‐limiting steps, specifically, the elementary steps involving the hydrogenation of CO_2_ to *COOH or *HCOO, and their subsequent hydrogenation to CO or HCOOH, respectively. The corresponding free‐energy landscapes for the complete reaction pathways, including water formation and desorption steps, are presented in Figure S1 (Supporting Information).

Figure 2 shows the ground‐state energy and grand‐potential profiles predicted from KS‐DFT and hybrid DFT, respectively. For each reaction pathway, the initial values are different because KS‐DFT describes the ground‐state energy (*E*) at 0 K, which is set to zero for the initial state of the catalyst surface bound with a single CO_2_ molecule (*CO_2_), while the hybrid DFT describes the grand potential of the entire heterogeneous system relative to that of a pristine surface in contact with the same gas phase (final state in Figure 2). As discussed above, Ω_cDFT_ depends on the density profiles and chemical potentials of all gas species, as well as their interaction with the catalyst surface (viz., the external potentials). At the initial state, the grand potential takes positive values because CO_2_ adsorption displaces gas‐phase species pre‐existing at the pristine catalyst surface, reducing the gas interaction with the metal surface. Moreover, at a given pressure, lowing the temperature increases the surface densities of gas‐phase molecules, thereby amplifying the energy penalty associated with such displacement.

Figure 2a shows that, in the pathway leading to CO formation, the rate‐determining step is the addition of the first hydrogen atom to the surface‐bound CO_2_, leading to the carboxyl intermediate (*COOH). After accounting for the catalyst interactions with gas‐phase molecules, the activation barrier increases compared to that predicted by KS‐DFT, making this step more difficult. The grand‐potential barrier (Ω_b_) shows a nonmonotonic temperature dependence, increasing from 300 K to 500 K, but decreasing from 500 K to 700 K. The underlying mechanism of this behavior may result from the redistribution of gas molecules near the active site, which will be further explored in the following section.

Figure 2b,c show the energy profiles for the reaction via the carboxyl (*COOH) and the formate (*HCOO) routes, respectively, leading to HCOOH formation. In contrast to the carboxyl route, the activation barrier for the initial step of CO_2_ hydrogenation increases monotonically with temperature. Additionally, the grand‐potential barrier predicted by the hybrid DFT is lower than the KS‐DFT energy barrier, suggesting that the reaction environment favors *HCOO formation. Because of the grand‐potential corrections to account for the microenvironmental effects, the hybrid DFT predicts a much smaller difference in the activation barrier between the *COOH and *HCOO pathways in comparison to that predicted by KS‐DFT. For example, the energy difference is reduced from −3.23 to −0.67 kcal mol^−1^ at 300 K. By applying the transition state theory (TST) to calculate the reaction rates for the two pathways, the rate ratio ($r_{*COOH}/r_{*HCOO}$) decreases from 225.6 to 3.1, raising the possibility that the *HCOO pathway is also kinetically competitive. Compared to the dominance of the carboxyl route predicted by KS‐DFT, the hybrid DFT prediction better aligns with experimental observations.^[^ ^36^, ^37^, ^38^, ^39^ ^]^

Figure 2 also presents the energy profiles for the subsequent elementary steps: *COOH → *CO + *OH, *COOH → *HCOOH, and *HCOO → *HCOOH. The hybrid DFT predicts that the presence of gas‐phase species consistently raises the activation barriers. Notably, in all three cases, the microenvironmental effect exhibits a nonmonotonic temperature dependence: the grand‐potential barrier increases from 300 K to 500 K, but decreases from 500 K to 700 K. This trend appears counterintuitive, as one might expect that with increasing temperature, the density of gas‐phase species at a given pressure should decrease, thereby weakening their interactions with surface‐bound intermediates. However, the grand potential reflects a more complex interplay of gas‐phase molecular distribution, surface structure, and configurational entropy, which together modulate the free‐energy barrier in a nonmonotonic manner.

It is worth noting that, for each elementary reaction, the grand‐potential correction (Ω_cDFT‐b_) to the activation energy is relatively small compared to that predict by KS‐DFT, typically on the order of a few kcal/mol. In contrast, the grand‐potential correction of the adsorption energy (Ω_cDFT‐ad_, see Equation 7) can exceed 10 kcal mol^−1^ for CO_2_ bounding with the catalyst surface. A similar correction applies to for the desorption energies of intermediates/products such as CO and HCOOH. The microenvironmental effect becomes more pronounced at low temperature because, in the presence of other gas species near the catalyst surface, chemically adsorption or desorption of a molecule induces substantial perturbations in the local densities of gas‐phase molecules, resulting in strong variations in the grand potential. These findings suggest that larger configurational changes in surface species lead to greater shifts in the grand potential due to catalyst‐microenvironment interactions.

### 2.2 Microenvironmental Effects on Reaction Mechanism

**Figure 3:**
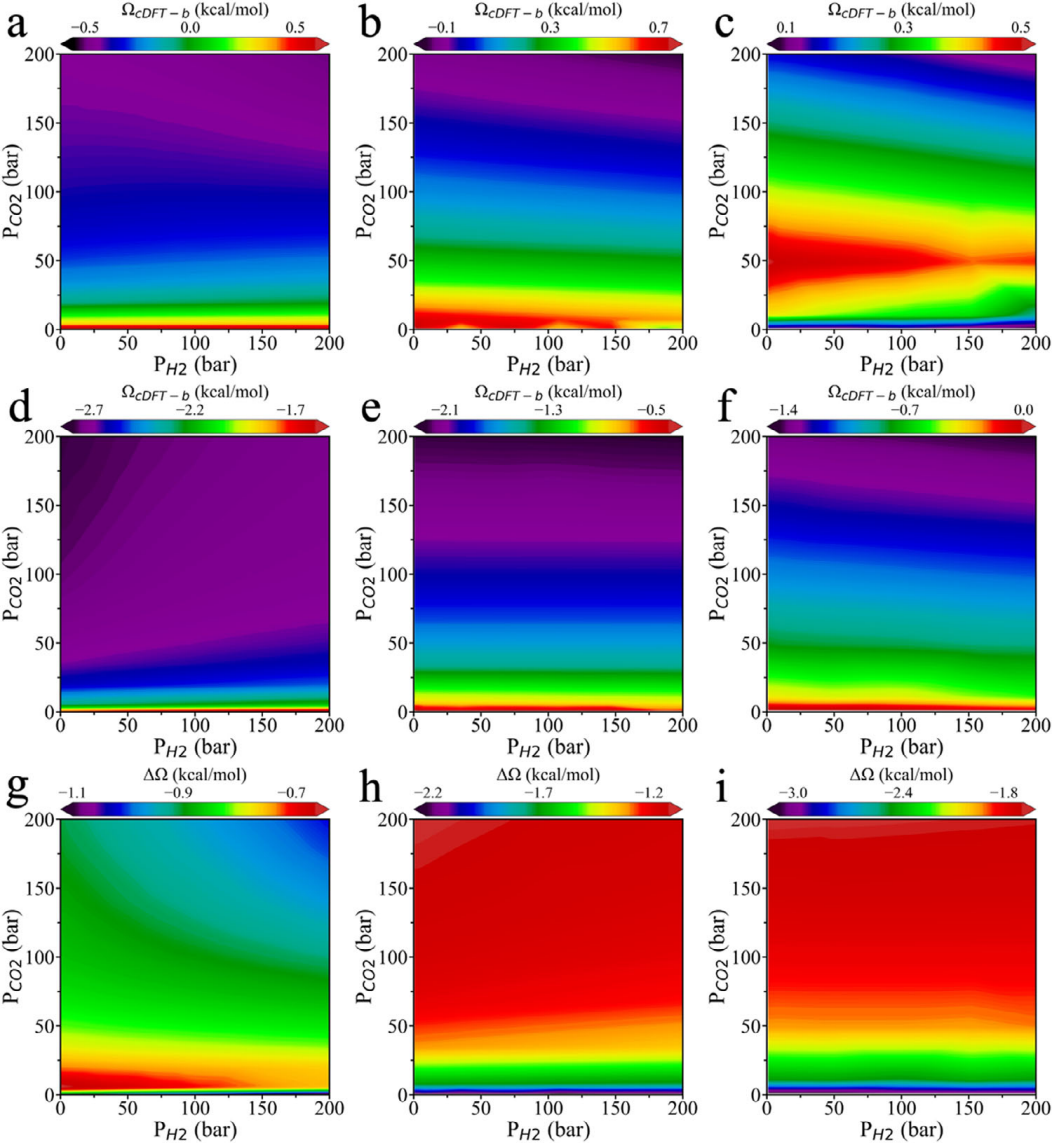
Dependence of the grand‐potential barriers on the partial pressures of H_2_ and CO_2_ in the gas phase. a–c) Grand‐potential correction to the key activation energy barrier (Ω_cDFT‐b_) in the COOH pathway of CO_2_ hydrogenation at 300 K (a), 500 K (b), and 700 K (c). d–f) Grand‐potential correction to the key activation energy barrier (Ω_cDFT‐b_) in the HCOO pathway of CO_2_ hydrogenation at 300 K (d), 500 K (e), and 700 K (f). g–i) Grand‐potential difference between the transition states of the two pathways (ΔΩ = Ω_TS‐COOH_ – Ω_TS‐HCOO_) at 300 K (g), 500 K (h), and 700 K (i).

To clarify microenvironmental effects on activation barriers, we focus again on the rate‐determining steps in two distinct CO_2_ hydrogenation pathways: *CO_2_ + *H → *COOH and *CO_2_ + *H → *HCOO. Specifically, we evaluated the grand‐potential corrections to their activation energy barriers (Ω_cDFT‐b_) at 300 K, 500 K, and 700 K over a wide range of partial pressures (P_CO2_ and P_H2_ varying from 0 to 200 bar). The results are shown in **Figure** **3**. Across all investigated temperatures, the partial pressure of CO_2_ (P_CO2_) plays a dominant role in modulating the grand‐potential corrections for both elementary reactions. Except for the case of *HCOO formation at 300 K (Figure 3d), the grand‐potential correction falls with increasing P_CO2_ and P_H2_, indicating that elevated pressures stabilize the transition states and facilitate the hydrogenation reactions. Besides, we observe that increasing temperature tendsz to destabilize the transition states, which is evident in the reduction of the grand‐potential correction for all paths.

Figure 3c shows that, at low pressures, Ω_cDFT‐b_ exhibits a non‐monotonic pressure dependence for the elementary reaction *CO_2_ + *H → *COOH. Ω_cDFT‐b_ starts with a positive value at low pressure, increases to a maximum, then falls toward zero, and eventually becomes negative (Figure 3a–c). Notably, the pressure at which this maximum occurs shifts to lower values as temperature decreases, with the peak already appearing below 1 bar at 300 K (Figure 3a). In contrast, for the *HCOO pathway (Figure 3d–f), the grand potential correction is all negative at low pressure and decreases further with increasing pressure.

Figure 3g–i present the difference in grand potential between the transition states of the two pathways (ΔΩ = Ω_*TS‐COOH_ – Ω_*TS‐HCOO_). The theoretical results reveal that, under all conditions investigated, the COOH pathway remains the more favorable route. Notably, at 300 K and around P_CO2_ = 10 bar, the grand potential difference reaches a minimum of ≈−0.6 kcal mol^−1^, indicating a near‐competitive scenario between the two pathways. Surprisingly, the pressure dependence of this grand potential difference varies significantly with temperature. At 300 K, increasing pressure further stabilizes the *TS‐COOH transition state, making ΔΩ more negative and thus favoring the COOH pathway. In contrast, at 500 K and 700 K, increasing pressure reduces the magnitude of the negative ΔΩ, thereby favoring the HCOO pathway. This temperature‐dependent pressure response represents a novel and intriguing finding: the effect of pressure on reaction selectivity reverses as temperature changes.

To rationalize the dominant effect of P_CO2_ on the reaction pathways, we defined the CO_2_ surface density as its average number density within 6 Å from the catalyst surface, aligned with the first peak in the local CO_2_ density profile. Figure S2 (Supporting Information) presents the surface densities of CO_2_ at the transition states of the rate‐limiting reactions over a wide range of gas conditions. As reported in our earlier work,^[^ ^41^ ^]^ the surface density differs significantly from the bulk gas density, often larger by several orders of magnitude. In all cases, the surface density increases sharply with rising P_CO2_, then gradually levels off as it approaches saturation. In contrast, the surface density of H_2_, also measured within 6 Å from the catalyst surface, increases gradually with P_H2_, and remains significantly lower than that of CO_2_ at all conditions (Figure S3a,e,I, Supporting Information). The stark difference in surface density explains why P_CO2_ has a dominant impact on the grand‐potential barriers. Furthermore, CO_2_ and H_2_ exhibit a competitive relationship at the catalyst surface: high P_CO2_ significantly suppresses the surface density of H_2_, while high P_H2_ also slightly reduces surface CO_2_ density. At 300 K, this mutual competition reduces the grand‐potential barrier, thus favoring *HCOO formation under high‐pressure conditions.

**Figure 4:**
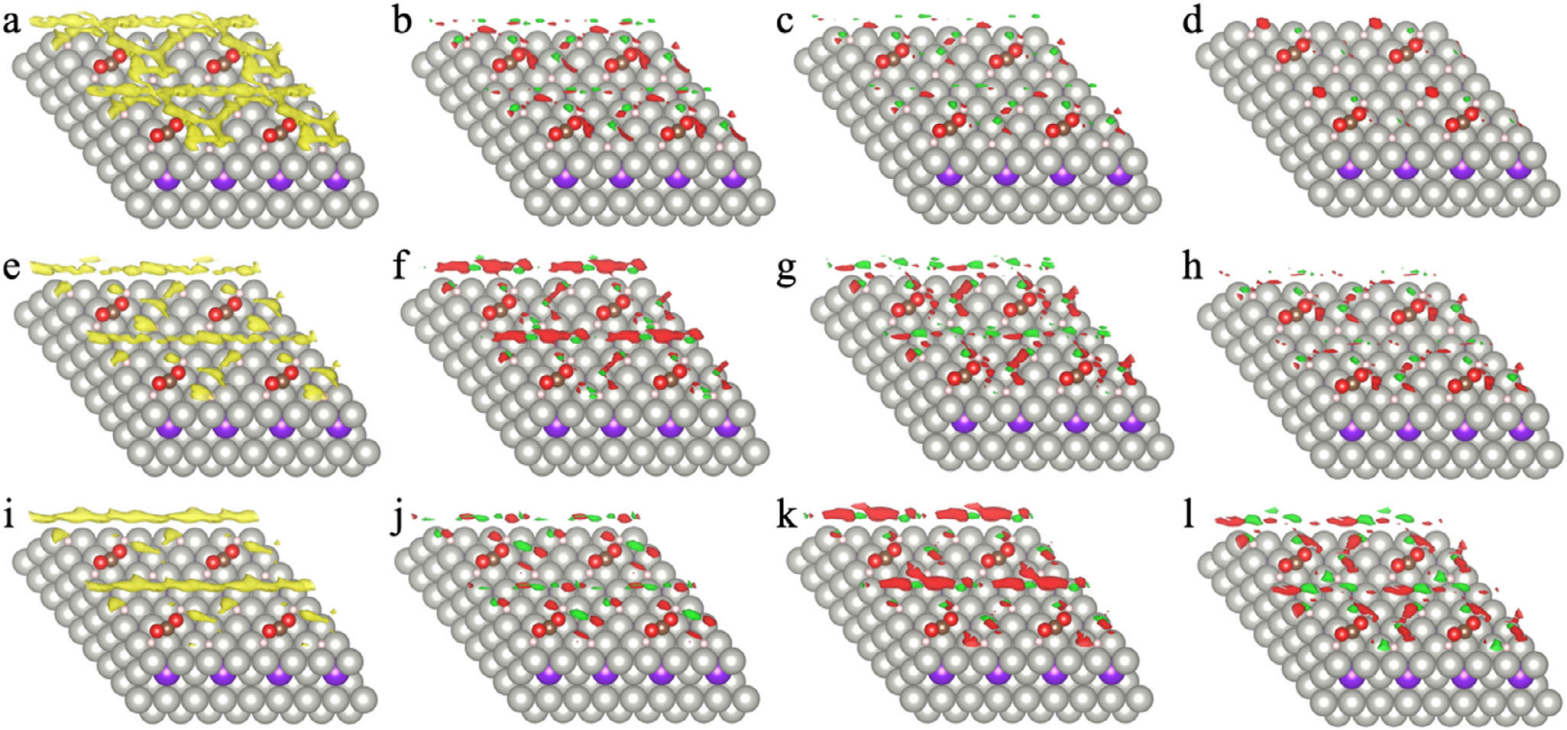
3D density maps for CO_2_ distribution near the catalyst surface at the transition state of elementary reaction *CO2 + *H → *COOH under different temperatures and bulk CO_2_ densities. a–d) *T*= 300 K; e‐h: *T*=500 K; i‐l: *T*=700 K. (a) The iso‐density surface at 10^−6^ molecules Å^−3^ with a CO_2_ bulk density (ρ_bulk‐CO2_) of 10^−5^ molecules Å^−3^ (corresponding to 0.4 bar). (b) Differential density map showing the change as ρ_bulk‐CO2_ increases from 10^−5^ to 10^−4^ molecules Å^−3^. (c) Differential density map ρ_bulk‐CO2_ increases from 10^−4^ to 5×10^−4^ molecules Å^−3^. (d) Differential density map as ρ_bulk‐CO2_ increases from 5×10^−4^ to 8×10^−3^ molecules Å^−3^ (corresponding to 200 bar). e–h) Corresponding density maps at 500 K for ρ_bulk‐CO2_ of 10^−5^ (corresponding to 0.7 bar) (e), 10^−5^ →10^−4^ (f), 10^−4^ →5×10^−4^ (g), and 5×10^−4^ →3×10^−3^molecules Å^−3^ (200 bar) (h). i–l) Corresponding density maps at 700 K for ρ_bulk‐CO2_ of 10^−5^ (corresponding to 1 bar) (i), 10^−5^ →10^−4^ (j), 10^−4^ →5×10^−4^ (k), and 5×10^−4^→2×10^−3^molecules Å^−3^ (200 bar) (l). All simulations were performed with a fixed hydrogen bulk density (ρ_bulk‐H2_) of 10^−5^ molecules Å^−3^. In all cases, the iso‐density surface is set at 10^−6^ molecules Å^−3^. Red indicates density increase, while green indicates density depletion.

**Figure** **4** shows the CO_2_ density profile at the transition state of the elementary reaction *CO_2_ + *H → *COOH under different thermodynamic conditions. At constant pressure, the gas‐phase density declines at higher temperature near the catalyst surface (Figure 4a,e,i), which explains the increase of the grand potential of the gas phase (Ω_cDFT_), thereby destabilizing the transition states. To elucidate the variation in the magnitude and sign of Ω_cDFT_, we examined the differential density maps as the CO_2_ partial pressure increases. Figure 4b–d,f–h,j–l and Figure S3 (Supporting Information) present the evolution of gas‐phase molecular distributions around the initial and transition states of the *COOH pathway. We see from these figures that the increase in surface gas‐phase density with rising pressure is not simply a monotonic, uniform enhancement, but rather involves a redistribution of gas molecules near the catalyst surface. At low pressures, increasing pressure primarily enhances gas density in regions far from the active site (referred to as long‐range zones), whereas at high pressures, fluctuations in the gas density occur predominantly near the adsorbates (short‐range zones). This implies a fundamental distinction between long‐range and short‐range interactions between gas‐phase molecules and surface configurations. The observation that gas molecules preferentially occupy regions farther from the active site suggests that long‐range interactions are generally stronger than short‐range interactions. This counterintuitive effect is attributed to the fact that short‐range interactions involve rearrangement of gas‐phase densities, which are weakened by intermolecular repulsion. Moreover, the difference in long‐ and short‐range energies for the interaction between the initial and transition states varies across surface configurations. For the *COOH pathway, at low pressure, the interaction is dominated by long‐range contributions, and the initial state interacts more strongly with the gas environment than the transition state, resulting in a positive grand potential correction that increases with pressure. At higher pressures, short‐range interactions become dominant, and the transition state exhibits stronger interactions, causing the correction value to peak and then decrease with further pressure increases—eventually turning zero or negative. In contrast, for the *HCOO pathway, the grand potential correction is already negative at low pressure and decreases further with increasing pressure. This is because, in this case, both long‐ and short‐range interactions are stronger for the transition state than for the initial state.

Furthermore, as shown in Figure 4 and Figures S4–S6 (Supporting Information), with increasing pressure, the gas‐phase molecular density in the long‐range region (i.e., areas far from the active site) increases progressively until saturation. Beyond this point, gas molecules mainly redistribute in the short‐range region to accommodate additional gas‐phase species. To further interpret the relative strengths of long‐range and short‐range interactions between the gas phase and the initial and transition states—and to explain the pressure at which the maximum in Ω_cDFT_ occurs—we analyzed the difference in surface gas density between the transition state and the initial state. As illustrated in Figure S2 (Supporting Information), at low pressures, the sign of the density difference in the long‐range region reflects which configuration (initial or transition state) exhibits stronger long‐range interactions. At intermediate pressures, the density difference in the short‐range region becomes more indicative of which state dominates short‐range interactions. At very high pressures, the surface becomes saturated, and the density difference approaches zero. Specifically, for the *COOH pathway, the initial state is characterized by stronger long‐range interactions than the transition state, while the latter exhibits stronger short‐range interactions. As pressure increases, the negative contribution to the density difference from the long‐range region diminishes, while the positive contribution from the short‐range region first increases and then decreases. The total density difference, therefore, evolves from a negative minimum toward zero and subsequently becomes positive. The point at which the total density difference equals zero represents the balance between long‐ and short‐range contributions. Beyond this pressure, the density difference becomes dominated by short‐range interactions. However, because long‐range interactions are generally stronger than short‐range ones as discussed above, the pressure corresponding to the maximum value of Ω_cDFT_ occurs slightly above the point where the net density difference crosses zero. This explains the non‐monotonic pressure dependence observed in the grand potential correction. It is also important to note that the influence of temperature on Ω_cDFT_ is not solely attributed to changes in the molecular density of the gas phase at a fixed pressure. At different temperatures, the saturation densities in both the long‐range and short‐range regions vary, meaning that the surface density corresponding to the energetic extrema differs across temperatures.

Based on this mechanism, we analyzed the pressure‐dependent trends of the grand potential energy barriers for Cu surfaces, as reported in our previous work.^[^ ^41^ ^]^ For the Cu (111) surface, the hydrogenation energy barrier of HCOO* increases with pressure, indicating that both long‐range and short‐range interactions between the initial state and gas components are stronger than those for the transition state. In contrast, for the Cu (211) surface, the energy barrier for HCOO* hydrogenation decreases with increasing pressure, suggesting that the long‐range and short‐range interactions between the initial state and gas components are weaker than those for the transition state.

Based on the above discussion, we can better understand the difference in grand potential between the transition states of the two pathways (ΔΩ = Ω_*TS‐COOH_ – Ω_*TS‐HCOO_), as shown in Figure 3g–I and discussed above. The origin of this behavior is consistent with the interaction trends illustrated in Figure S2d,h,l (Supporting Information). At 700 K and low pressure, the surface gas‐phase density is extremely low, and the density difference is negative—indicating that the *TS‐HCOO transition state experiences stronger long‐range interactions with the gas phase. As pressure increases, the density difference becomes positive, signifying stronger short‐range interactions for the *TS‐COOH transition state. Consequently, at low surface gas densities, the grand potential difference starts from its KS‐DFT baseline (−3.23 kcal mol^−1^) and increases with pressure. At 300 K and ≈10 bar, this enhancement effect reaches its maximum. Beyond this point, short‐range interactions dominate and further increases in pressure reduce the grand potential difference.

**Figure 5:**
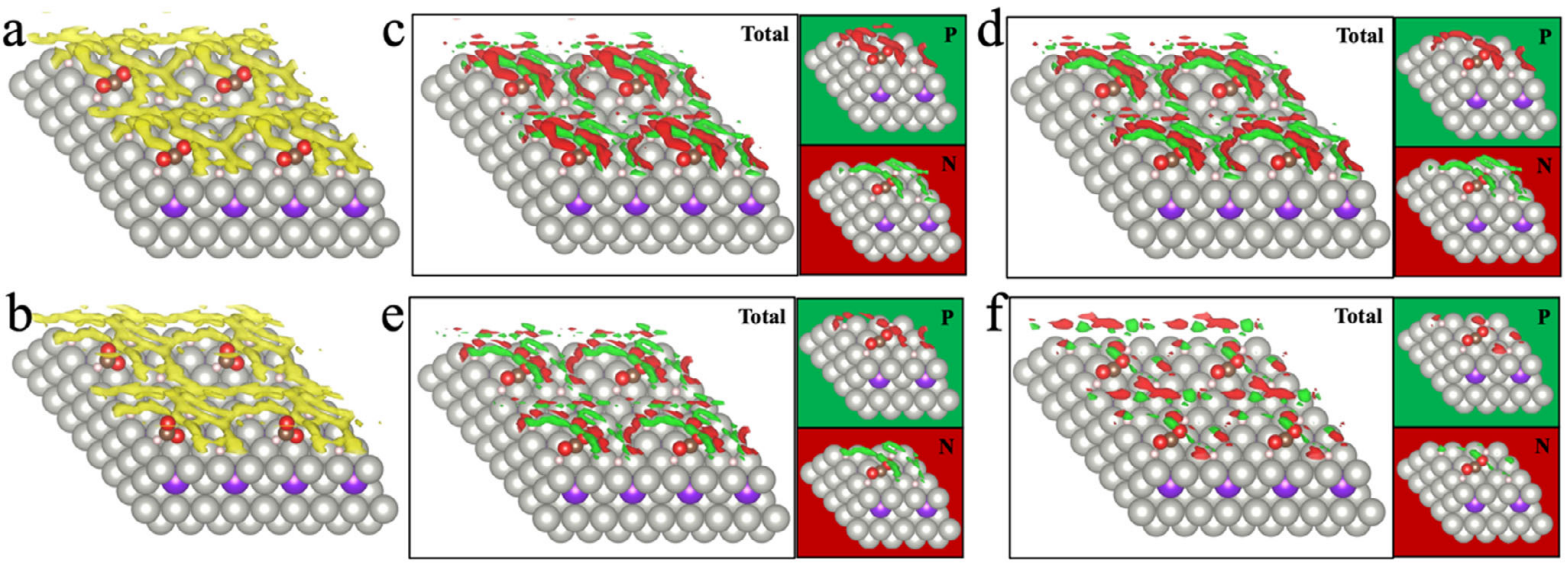
The local CO_2_ density near the catalyst surface at the transition states of two hydrogenation pathways. a) 3D gas‐density profile for the *COOH transition state at 300 K, ρ_bulk‐CO2_ = 10^−3^ molecules Å^−3^ (35 bar), ρ_bulk‐H2_ = 10^−3^ molecules Å^−3^ (42 bar). b) 3D gas‐density profile for the *HCOO transition state under the same conditions. c–f) Differential density maps between the *COOH and *HCOO transition states (ρ_TS‐COOH_ – ρ_TS‐HCOO_): (c) at 300 K with ρ_bulk‐CO2_ = 10^−3^ molecules Å^−3^ (35 bar), ρ_bulk‐H2_ = 10^−3^ molecules Å^−3^ (42 bar); (d) Differential density maps at 300 K with ρ_bulk‐CO2_ = 2.5×10^−4^ molecules Å^−3^ (10 bar), ρ_bulk‐H2_ = 7.5×10^−4^ molecules Å^−3^ (30 bar); (e) Differential density maps at 300 K with ρ_bulk‐CO2_ = 10^−5^ molecules Å^−3^ (0.4 bar), ρ_bulk‐H2_ = 4×10^−3^ molecules Å^−3^ (180 bar); (f) Differential density maps at 700 K with ρ_bulk‐CO2_ = 5×10^−4^ molecules Å^−3^ (50 bar), ρ_bulk‐H2_ = 5×10^−4^ molecules Å^−3^ (50 bar). All density maps use an iso‐surface value of 10^−6^ molecules Å^−3^. Red indicates density increase (Positive); green indicates density depletion (Negative).

It is important to note that the grand‐potential corrections of the energy barrier (ΔΩ_cDFT_ = Ω_cDFT‐TS‐COOH‐_ – Ω_cDFT‐TS‐HCOO_) are not solely determined by the difference in the surface density of CO_2_. As shown by the 3D configurational differential density maps in **Figures** **5** and S7 (Supporting Information), even slight changes in surface configuration can lead to substantial shifts in the local density distribution for CO_2_ molecules. While the surface density difference reflects the spatial variation in gas‐phase interactions between catalyst configurations, they do not capture the absolute local gas density surrounding each surface state. This overall increase in the local density upon pressurization is the primary factor contributing to the trend for the grand potential correction of the energy barrier.

### 2.3 Effects of the Surface Structure

To investigate the coupling effects of the reaction environment and the catalytic structure, we examined the reaction pathways on the PdZn catalyst with a higher hydrogen coverage (PdZn_H075, 0.75 adsorbed hydrogen coverage). Previous work by Zhang and Liu.^[^ ^34^ ^]^ reported that the PdZn alloy exhibits different hydrogen coverages under varying conditions of temperature and pressure, altering the CO_2_ activation energy and product selectivity. The KS‐DFT calculations indicate that the increased H coverage leads to significantly larger differences in activation barriers among the three reaction pathways, making the COOH to HCOOH pathway more dominant. As shown in Figure S8 (Supporting Information), under these conditions, the influence of environmental (gas‐phase) effects on reaction selectivity is minimal, as the grand potential corrections to the transition states (Ω_cDFT‐b_) are less than 3 kcal mol^−1^, substantially smaller than the energy differences between pathways predicted by KS‐DFT alone. Moreover, the Ω_cDFT_ corrections are generally smaller for PdZn_H075 than the PdZn_H025 under the same temperature and pressure conditions. For the CO_2_ → *COOH pathway, Ω_cDFT‐b_ takes the values of −0.93, 0.11, and 0.11 kcal mol^−1^ at 300, 500, and 700 K, respectively. For the CO_2_ → *HCOO pathway, the corresponding Ω_cDFT‐b_ values are −1.49, −0.27, and 0.20 kcal mol^−1^. In addition to the reduction of magnitude, the surface coverage alters both the sign of Ω_cDFT‐b_ and its temperature‐dependent trend. This change is likely attributed to the variation in gas‐phase surface density induced by differences in the surface coverage of H.

**Figure 6:**
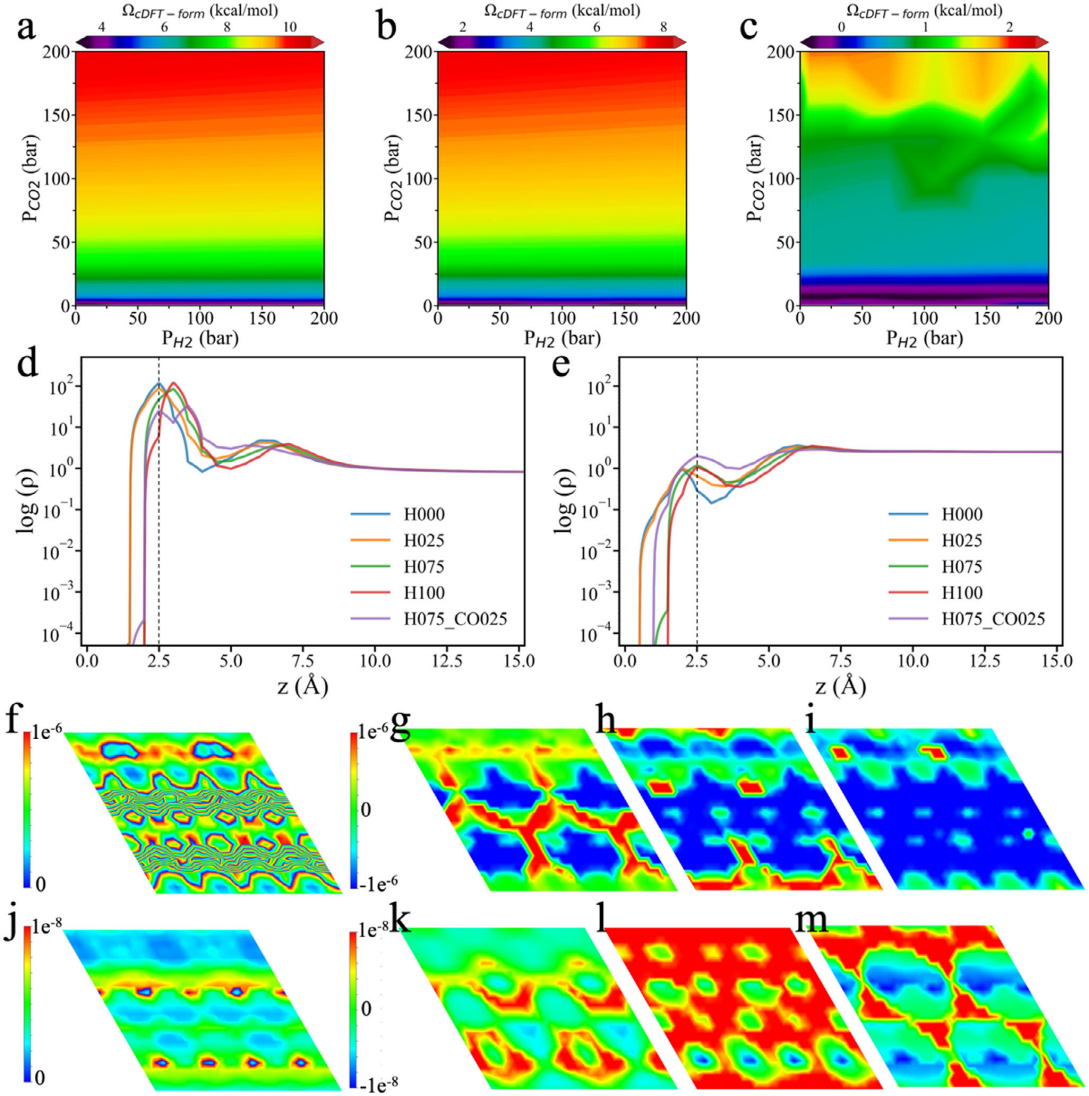
Variation of the grand potential and gas density profile with H surface coverage. a–c) Grand potential correction for the formation of the *COOH intermediate (Ω_cDFT‐form_) as a function of gas‐phase partial pressures for CO_2_ hydrogenation on PdZn_H025 (a), PdZn_H075 (b), and PdZn_H075_CO025 (c). d) 1D CO_2_ density profile as a function of distance from the surface (z= 0 denotes the surface). e) 1D H_2_ density profile as a function of the normal distance from the surface. f) 2D CO_2_ density profile at 2.5 Å above the surface for PdZn_H000 (no H coverage). g–i) 2D differential density maps of CO_2_ comparing PdZn_H025 (g), PdZn_H075 (h), and PdZn_H075_CO025 (i) to PdZn_H000. j–m) Corresponding differential density maps for H_2_. All results were obtained at 500 K under gas‐phase conditions of P_CO2_ = 10 bar and P_H2_ = 30 bar for panels d–m.

To systematically investigate how surface coverage affects the interactions between gas‐phase components and reaction intermediates, we studied the grand potential corrections to the formation energy (Ω_cDFT − form_, see Equation 2) for each surface structure. **Figures** **6**a–c and S9 (Supporting Information) present the results for both *COOH and *HCOO configurations on surfaces with varying coverages, including PdZn_H000, PdZn_H025, PdZn_H075, PdZn_H100, and PdZn_H075_CO025 (where the numeric subscripts denote the fractional surface coverages of different adsorbates). As discussed above, Ω_cDFT‐form_ reflects the grand‐potential contribution to the free energy of formation, arising from changes induced by the occupation of surface intermediates. For all surfaces, Ω_cDFT‐form_ is predominantly influenced by the CO_2_ partial pressure, which is consistent with previous analyses (Figure S2, Supporting Information). Additionally, it is noteworthy that for the PdZn_H075_CO025 surface (with 0.75 monolayers of H and 0.25 monolayers of CO), the influence of hydrogen partial pressure on Ω_cDFT‐form_ becomes more pronounced, indicating that surface composition can modulate the relative sensitivity to different gas‐phase components. When comparing across surfaces with different coverage levels, a clear trend emerges: Ω_cDFT‐form_ decreases significantly with increasing hydrogen coverage and becomes even smaller in the presence of co‐adsorbed CO species. This observation indicates that as surface coverage increases—particularly with larger adsorbates—the energy arising from interaction between gas‐phase molecules and surface intermediates is reduced due to enhanced steric hindrance and limited gas accessibility near the active sites.

To elucidate the mechanism behind the different grand potential contributions observed across different surfaces with varying coverages, we also analyzed the gas‐phase molecular density distributions on these surfaces. The 1D density profiles of CO_2_ gas molecules shown in Figure 6d reveal that this reduction in the free‐energy barrier can be attributed to a smaller total CO_2_ gas density near the catalyst as the surface coverage of H increases. However, due to its smaller molecular size, H_2_ exhibits an increased local density near the catalyst surface (Figure 6e), in contrast to the more substantial decline of the local density for CO_2_. To further illustrate these findings, we presented the 2D gas‐phase density maps at 2.5 Å from the surface in Figure 6f,j and Figure S10 (Supporting Information), alongside the differential density maps under different surface coverages (Figure 6g–i,k–m; Figure S11, Supporting Information). These density maps clearly illustrate the spatial exclusion of gas molecules caused by surface‐bound species and the accumulation of gas molecules at surface edges. Notably, the reduction in the gas density is more pronounced for larger molecules like CO_2_, whereas smaller molecules like H_2_ show a greater increase in the surface density. These results indicate that as surface coverage increases, the influence of large gas‐phase molecules on catalytic energetics diminishes due to steric hindrance, while the role of gas‐phase species of smaller size becomes more important. Consequently, Ω_cDFT‐form_ decreases with higher surface coverage, and after CO co‐adsorption, the impact of hydrogen partial pressure becomes more significant.

Additionally, we analyzed the impact of gas‐phase byproduct H_2_O on the CO_2_ hydrogenation barriers for both surface coverages. The results are shown in Figure S12 (Supporting Information). When a small amount of water is present in the gas phase, the hydrogenation barriers for both pathways on both surfaces decrease. This is because, even with a small amount of water, the strong attraction between the catalyst surface and H_2_O leads to a sharp increase in the H_2_O surface density. This density can even exceed the CO_2_ surface density, allowing H_2_O to dominate the surface adsorption and reduce the energy barrier. As the partial pressure of H_2_O continues to increase, the H_2_O surface density grows more slowly, accompanied by a decrease in the CO_2_ surface density. The former lowers the energy barrier, while the latter increases the barrier, and their combined effects lead to a non‐monatomic dependence of the energy barrier on H_2_O partial pressure.

## 3 Conclusion

In this work, we investigated the environmental effects on CO_2_ hydrogenation over PdZn alloy surfaces with varying surface coverages. By integrating KS‐DFT with classical DFT under a grand canonical ensemble, we incorporated the effects of gas‐phase composition, pressure, and temperature through probability density distributions of gas molecules—offering a more realistic depiction of catalytic environments beyond traditional vacuum‐based models. Our results reveal that gas‐phase molecules can exert opposite influences on different reaction pathways, with their impact strongly modulated by temperature and pressure. Notably, we identified a temperature‐dependent shift in selectivity: while increased pressure favors the COOH pathway at low temperatures, it enhances the competitiveness of the HCOO pathway at higher temperatures.

Through multidimensional gas‐density analyses—including 1D, 2D, and 3D distributions as well as pressure‐ and configuration‐resolved differential maps—we disentangled the roles of long‐range and short‐range interactions between gas molecules and surface intermediates, and revealed the heterogeneous spatial response of gas‐phase species near the catalyst surface. We also demonstrated that increasing surface coverage suppresses the environmental effect of larger gas molecules (e.g., CO_2_), while enhancing the influence of smaller species like H_2_, due to steric exclusion and edge‐induced redistribution. Together, these findings provide a mechanistic understanding of how catalytic surface structure and reactive gas environments interact to shape reaction energetics and selectivity. This study offers a refined strategy for incorporating environmental factors into first‐principles modeling, with implications for the rational design of catalysts under realistic operating conditions.

## 4 Computational Method

4.1

The computational box was divided into two subsystems: one includes the catalyst with chemically adsorbed species, and the other consists of the environmental gas molecules. The two subsystems were decoupled through surface‐gas interactions. The grand potential of the entire system (Ω) was calculated from:

(1) $$\Omega =E_{\mathrm{KS}-\mathrm{DFT}}+\Omega_{\mathrm{cDFT}}$$

where E_KS − DFT_ represent the ground‐state energy of the catalyst and surface species, and Ω_cDFT_ represents the grand potential of the environmental gas molecules. While E_KS − DFT_ was obtained from KS‐DFT calculations, Ω_cDFT_ was calculated using cDFT, which also provides the density profiles of all gas‐phase components around the catalyst surface at a specified temperature and the chemical potentials of individual chemical species.

It is important to note that in constructing energy profiles, the absolute value depends on the choice of a reference state. In this work, the KS‐DFT energy profiles were referenced to the CO_2_ adsorption configuration, where the energy of the catalyst with adsorbed *CO_2_ is set to zero. In contrast, the grand potential profiles were expressed relative to that of the pristine surface (i.e., catalyst without any adsorbate) physically in contact with CO_2_ and H_2_ molecules from the gas phase. In other words, all Ω_cDFT_ values were calculated relative to the grand potential of the pristine reference system at the same bulk condition. The grand potential correction thus reflects the change in the external potential of the gas phase resulting from structural variations at the surface. This correction leads to a variation of the formation (free) energy for each intermediate state and is defined as:

(2) $$\begin{array}{ccc} \Omega_{cDFT-form} & = & \Omega_{\mathrm{cDFT}-*\mathrm{state}}-\Omega_{\mathrm{cDFT}-*}-(n\Omega_{\mathrm{cDFT}-\mathrm{CO}2}\\ & & +\;m\Omega_{\mathrm{cDFT}-H2}-\Omega_{cDFT-bulk}) \end{array}$$

where Ω_*cDFT* − *state_, Ω_*cDFT* − *_, Ω_*cDFT* − CO2_, and Ω_*cDFT* − H2_are the grand potentials of the gas phase in contact with the catalyst surface bounded with an intermediate state (*‐state), the gas phase in contact with the pristine surface without chemisorption (*), the gas molecules of CO_2_ and H_2_ surrounded by the gas molecules in the bulk (viz., without surface), respectively; n and m are the numbers of CO_2_ and H_2_ molecules associated with the intermediate state (*‐state), and Ω_*cDFT* − *bulk*_ is the grand potential of the bulk gas‐phase system. Since the cDFT simulation was performed under the µVT ensemble, it requires the system temperature, total volume, and the chemical potentials of all gas species to remain constant.

The cDFT simulations were carried out using the in‐house developed GPU‐accelerated cDFT package, assuming that gas molecules can be represented by the Lennard‐Jones (LJ) model.^[^ ^42^ ^]^ It has been demonstrated in the previous work that the cDFT method was both highly efficient and accurate in simulating gas adsorption in nanoporous materials.^[^ ^43^, ^44^ ^]^ Briefly, the thermodynamic properties of gas molecules near a catalytic surface are derived from the grand potential:

(3) $$\Omega_{\mathrm{cDFT}}=\;F\left[\rho_{i}\left(r\right)\right]+\sum_{i}\int \left[V_{i}^{\mathrm{ext}}\left(R,r\right)-\mu_{i}\right]\rho_{i}\left(r\right)dr$$

where F[ρ_i_(**r**)] represents the intrinsic Helmholtz energy of the gas phase, and $V_{i}^{\mathrm{ext}}\left(R,r\right)$ is the external potential of gas species i, and µ_i_ is the chemical potential.

In this work, the external potential corresponds to the energy experienced by each gas molecule due to its interaction with the catalyst surface (viz., with both metals and adsorbates). More specifically, $V_{i}^{\mathrm{ext}}\left(R,r\right)$ describes the interaction of a gas molecule at position **r** with the catalyst and chemically adsorbed species with atomic configuration **R**. The external potential experienced by each gas molecule due to its interaction with a surface atom is also given by the LJ potential.

(4) $$V_{LJ}\left(r\right)=4\varepsilon \left[{\left(\frac{\sigma }{r}\right)}^{12}-{\left(\frac{\sigma }{r}\right)}^{6}\right]$$

where ε and σ are the energy and size parameters, and *r* is the center‐to‐center distance between the gas molecule and the surface atom. The LJ potential parameters, including those for gas‐gas interactions, are listed in Table S1 (Supporting Information). The Lorentz‐Berthelot mixing rules were used for describing the LJ interactions between different species. To calculate the chemical potential of each species, the modified Benedict–Webb–Rubin (MBWR) equation of state was utilized, which was well‐suited for bulk gas‐phase systems under various conditions studied in this work.^[^ ^45^ ^]^

After obtaining the grand potential (Ω) for each reaction intermediate, the reaction energy barrier in the grand potential framework (Ω_b_) was calculated based on the following equation:

(5) $$\Omega_{b}=\Omega_{\mathrm{TS}}-\Omega_{\mathrm{IS}}=E_{b}+\Omega_{\mathrm{cDFT}-b}$$

where E_b_ = E_TS_ − E_IS_ corresponds to the difference between the ground‐state energies of the isolated surface at the transition state (TS) and the initial state (IS) of the reaction, the grand potential correction term, Ω_cDFT − b_, is defined as:

(6) $$\Omega_{\mathrm{cDFT}-b}=\Omega_{\mathrm{cDFT}-\mathrm{TS}}-\Omega_{\mathrm{cDFT}-\mathrm{IS}}$$

Compared to the energy barrier (E_b_) obtained from conventional KS‐DFT calculations, the grand potential barrier (Ω_b_) includes an additional correction term, Ω_cDFT − b_, accounting for the effect of the surface interaction with the surrounding environment.

Similar to the environmental correction of the formation energy (Ω_*cDFT* − *form*_), the gas phase was explicitly considered in calculating the adsorption energy. In this case, both the pristine catalyst and the catalyst with the adsorbates were influenced by gas molecules in the bulk phase. The grand‐potential correction to the adsorption energy is thus given by

(7) $$\begin{array}{c} \Omega_{cDFT-ad}=\Omega_{\mathrm{cDFT}-*\mathrm{adsorbate}}-\Omega_{\mathrm{cDFT}-*}-\Omega_{\mathrm{cDFT}-\mathrm{adsorbate}}+\Omega_{cDFT-bulk} \end{array}$$

where Ω_*cDFT* − *adsorbate_, Ω_*cDFT* − *_, Ω_*cDFT* − adsorbate_ and Ω_*cDFT* − *bulk*_ are the grand potentials of the gas phase in contact with the surface with adsorbate (*adsorbate), the gas phase in contact with the pristine surface without chemisorption (*), the adsorbate surrounded by the gas molecules in the bulk (viz., without surface), and the bulk gas phase, respectively.

## Conflict of Interest

The authors declare no conflict of interest.

## Supporting information



Supporting Information

## Data Availability

The data that support the findings of this study are available from the corresponding author upon reasonable request.
