# Supplementary material for: Microenvironmental Effects on CO2 Hydrogenation Over PdZn Alloy Catalysts
Source: Adv Sci (Weinh). 2025 Sep 14;12(39):e09726. doi: 10.1002/advs.202509726 (PMC12533389; doi:10.1002/advs.202509726)
Supplement: Supplementary file 1 — Supporting Information [file ADVS-12-e09726-s001.docx]

Microenvironmental Effects on CO_2_ Hydrogenation over PdZn Alloy Catalysts

Jikai Sun, Jianzhong Wu *

Department of Chemical and Environmental Engineering, University of California, Riverside, CA 92521, USA

E-mail: jianzhong.wu@ucr.edu

The Supporting Information provides the model parameters (Table S1) and additional results (Figure S1-S11) from classical density functional theory (cDFT) calculations.

**Transition State Theory (TST)**

According to the transition state theory (TST)^[1]^, the rate constant $k_{i}$ for each surface reaction can be expressed as:

$k_{i}=\frac{k_{B}T}{h}e^{\frac{-\Delta G}{k_{B}T}}$ (1)

where $\Delta G$ represent the energy barrier after the correction for the bond vibration entropy of the substrate.

Two surface reactions were under consideration in this work:

CO_2_ + * + H == *COOH (R1)

CO_2_ + * + H == *HCOO (R2)

Since these two reactions are rate-limiting, only their forward reaction rates were considered. For each reaction, the reaction rate can be expressed as:

$r_{i}=k_{i,f}P_{{CO}_{2}}\theta_{*}P_{H}$ (2)

where $P_{{CO}_{2}}$ and $P_{H}$ are the partial pressures of CO_2_ and hydrogen atoms in the gas phase, respectively. Eq. (2) predicts that the rate ratio of these two reactions is:

$\frac{r_{1}}{r_{2}}=e^{\frac{-({\Delta G}_{1}-{\Delta G}_{2})}{k_{B}T}}=e^{\frac{-(G_{*COOH}-G_{*HCOO})}{k_{B}T}}$ (3)

where ${\Delta G}_{1}$ and ${\Delta G}_{2}$ represent the energy barriers for reactions R1 and R2, and $G_{*COOH}$ and $G_{*HCOO}$ represent the transition state energies for *COOH and *HCOO, respectively, with the initial state energies being canceled out. Within our hybrid quantum/classical framework, and using the grand potential rather than the Gibbs free energy, Eq. (3) becomes:

$\frac{r_{1}}{r_{2}}=e^{\frac{-({\Delta\Omega}_{1}-{\Delta\Omega}_{2})}{k_{B}T}}=e^{\frac{-(\Omega_{*COOH}-\Omega_{*HCOO})}{k_{B}T}}$ (4)

**Table S1** The Lennard-Jones parameters for gas molecules and surface atoms of the catalyst considered in this work.

| Species | ε (K) | σ (Å) | Ref |
| --- | --- | --- | --- |
| H_2_ | 36.7 | 2.96 | ^[2]^ |
| CO_2_ | 200.9 | 3.943 | ^[3]^ |
| H_2_O | 506.0 | 2.71 | ^[3]^ |
| Pd | 5319.3 | 2.5158 | ^[4]^ |
| Zn | 1966.6 | 2.4871 | ^[4]^ |
| C | 52.85 | 3.851 | ^[5]^ |
| H | 22.14 | 2.886 | ^[5]^ |
| O | 30.20 | 3.50 | ^[5]^ |

**
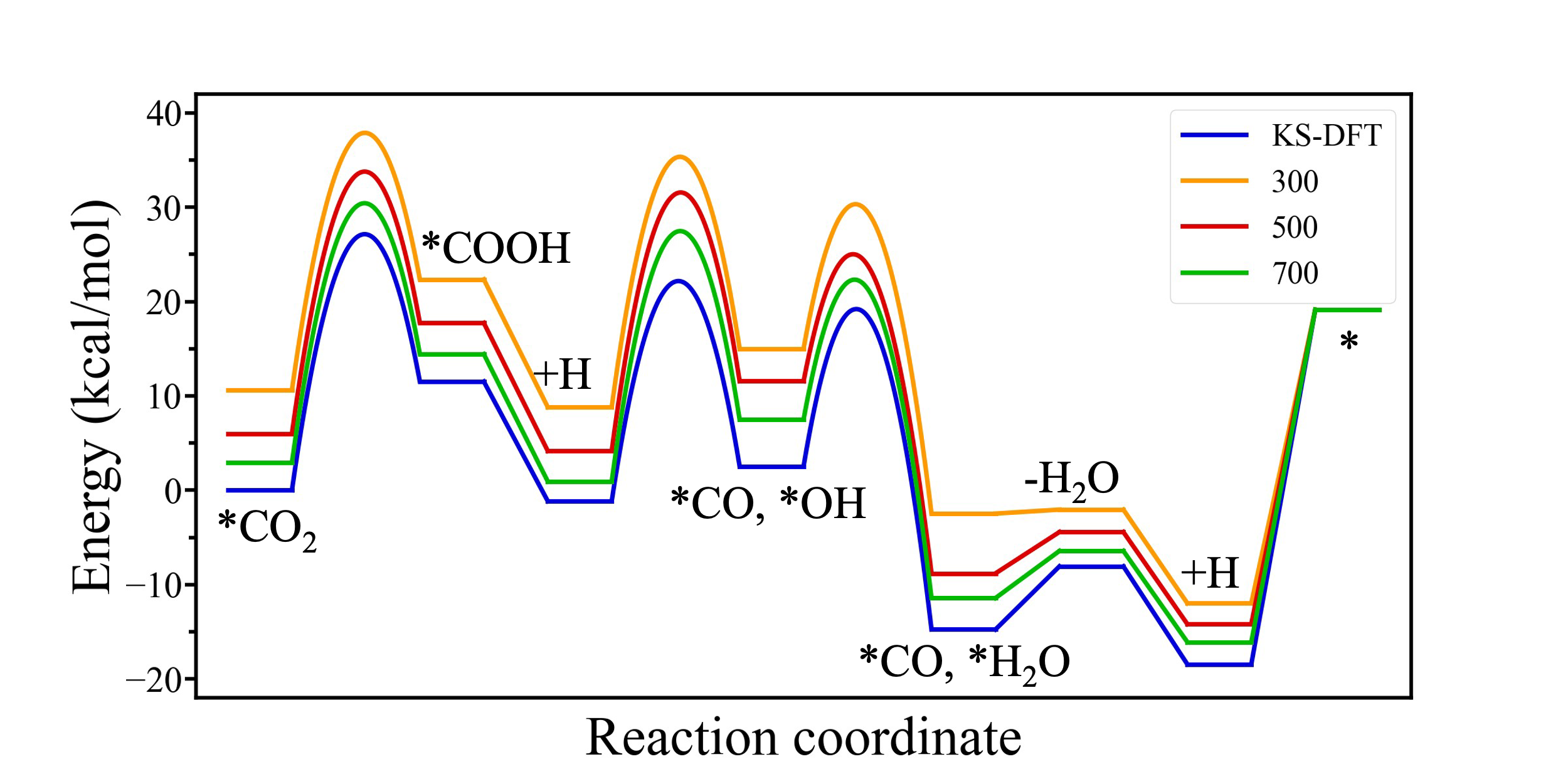
**

**Figure S1**. Complete reaction energy profile for CO_2_ hydrogenation to CO. The grand potential profiles were calculated under the gas-phase conditions of P_CO2_ = 10 bar and P_H2_ = 30 bar at temperatures of 300 K, 500 K, and 700 K.


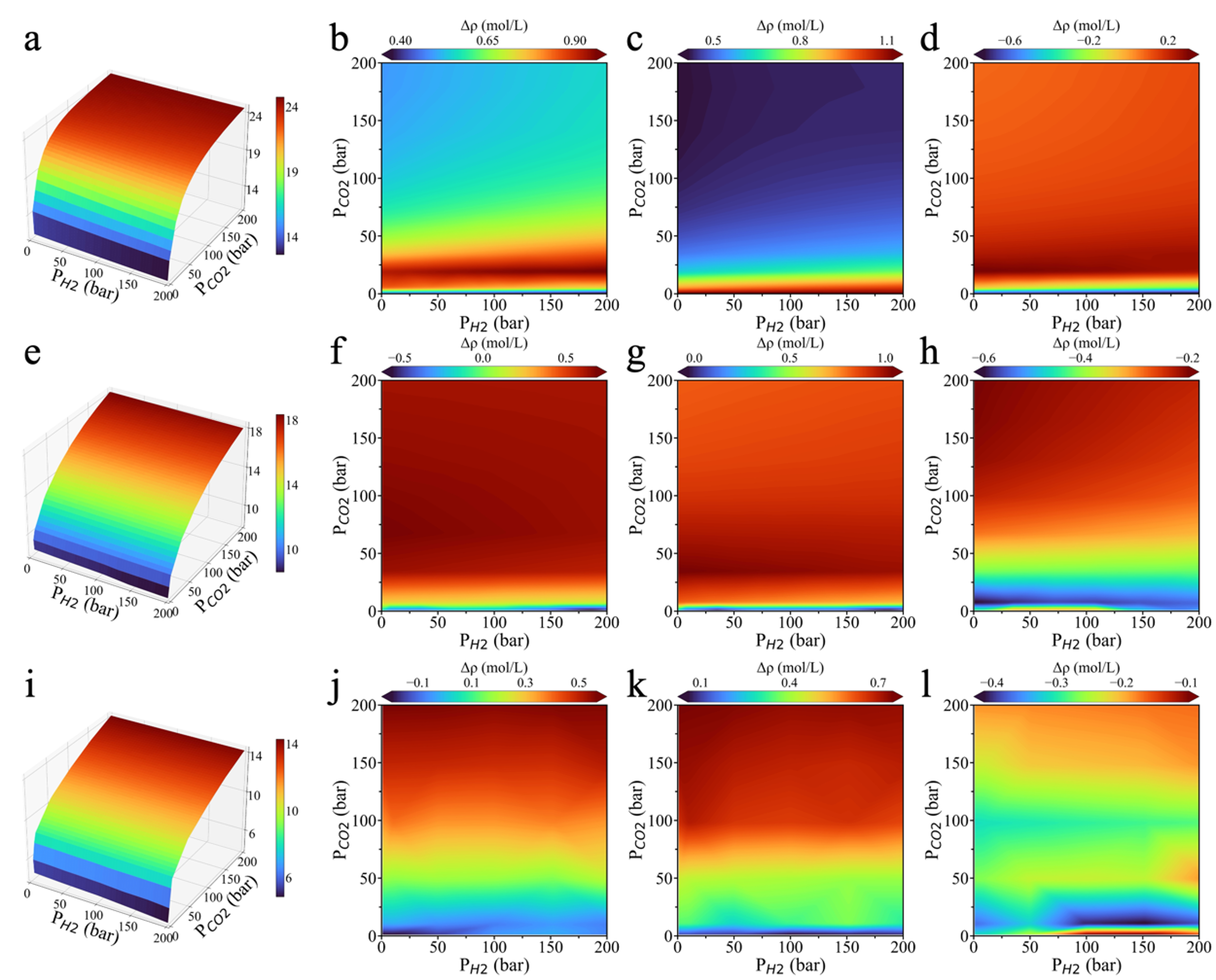


**Figure S2**. CO_2_ surface density as a function of the partial pressures of CO_2_ and H_2_ in the bulk phase. a. Surface density (ρ) at the transition state of the elementary reaction *CO_2_ + *H → *COOH at 300 K. b. Difference between the CO_2_ surface densities at the transition and initial states of the same elementary reaction (Δρ = ρ_TS_ – ρ_IS_). c. Surface density difference between the transition and initial states of the elementary reaction *CO_2_ + *H → *HCOO (Δρ = ρ_TS_ – ρ_IS_) at 300 K. d. Surface density difference between the transition states of the two elementary reactions (Δρ = ρ_TS-COOH_ – ρ_TS-HCOO_). e-h. The same as plots a-d but at 500 K. i-l. The same as plots a-d but at 700 K.


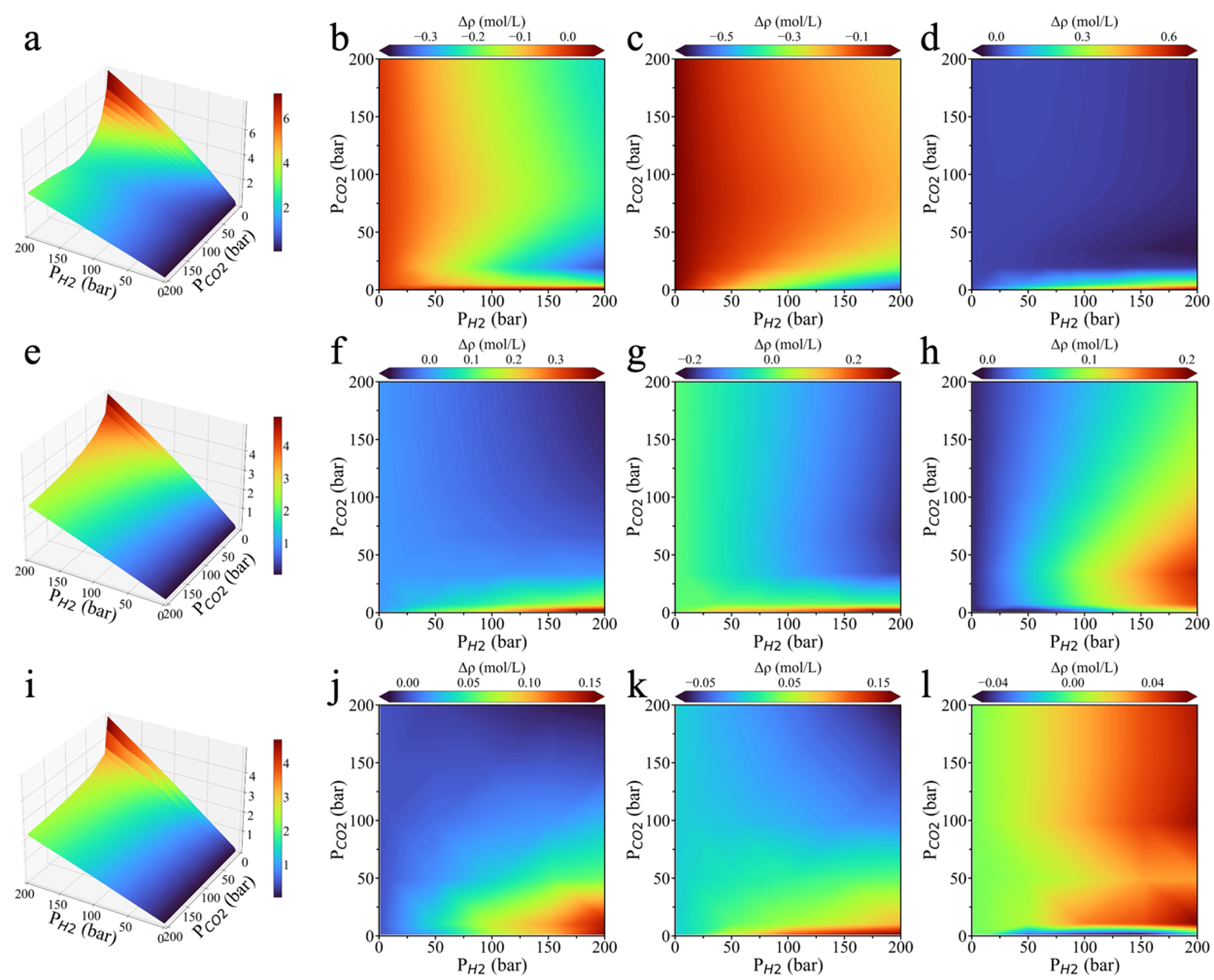


**Figure S3**. Surface density of H_2_ as a function of gas-phase partial pressures. a. Surface density (ρ) at the transition-state configuration of the *COOH pathway at 300 K. b. Surface density difference between the transition and initial states of the *COOH pathway (Δρ = ρ_TS_ – ρ_IS_) as a function of gas-phase partial pressures at 300 K. c. Surface density difference between the transition and initial states of the *HCOO pathway (Δρ = ρ_TS_ – ρ_IS_) at 300 K. d. Surface density difference between the transition states of the two pathways (Δρ = ρ_TS-COOH_ – ρ_TS-HCOO_) at 300 K. e-h. Corresponding plots at 500 K. i-l. Corresponding plots at 700 K.


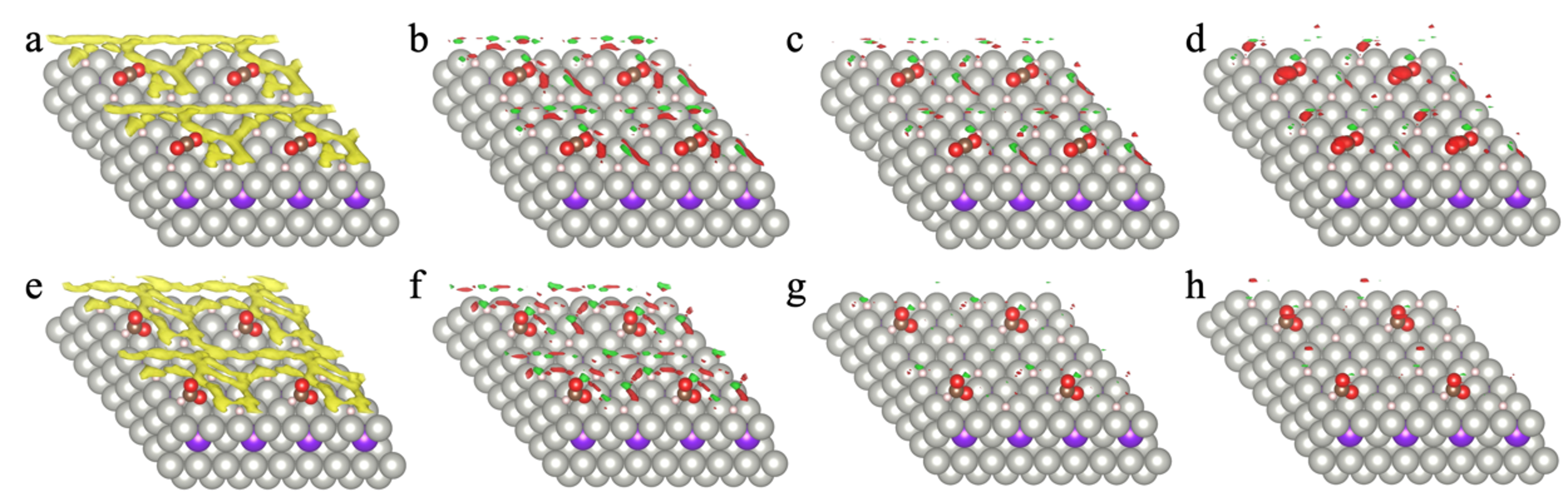


**Figure S4**. Three-dimensional (3D) density maps for CO_2_ distribution near the catalyst surface at the transition state of elementary reaction *CO2 + *H → *HCOO at 300 K under varying CO_2_ bulk densities. a. Density map at the initial CO_2_ adsorption configuration with ρ_bulk-CO2_ = 0.00001 molecules/Å^3^. b–d. Differential density maps showing changes as ρ_bulk-CO2_ increases from 0.00001 to 0.0001 (b), 0.0001 to 0.0005 (c), and 0.0005 to 0.008 molecules/ Å^3^ (180 bar) (d) for the initial state. E. Density map at the *HCOO transition state with ρ_bulk-CO2_ = 0.00001 molecules/ Å^3^. f–h. Differential density maps at the *HCOO transition state as ρ_bulk-CO2_ increases from 0.00001 to 0.0001 (f), 0.0001 to 0.0005 (g), and 0.0005 to 0.008 molecules/ Å^3^ (180 bar) (h). All simulations were conducted at 300 K with a fixed hydrogen bulk density of ρ_bulk-H2_ = 0.00001 molecules/ Å^3^. The isosurface value is set at 1×10^-6^ molecules/ Å^3^.


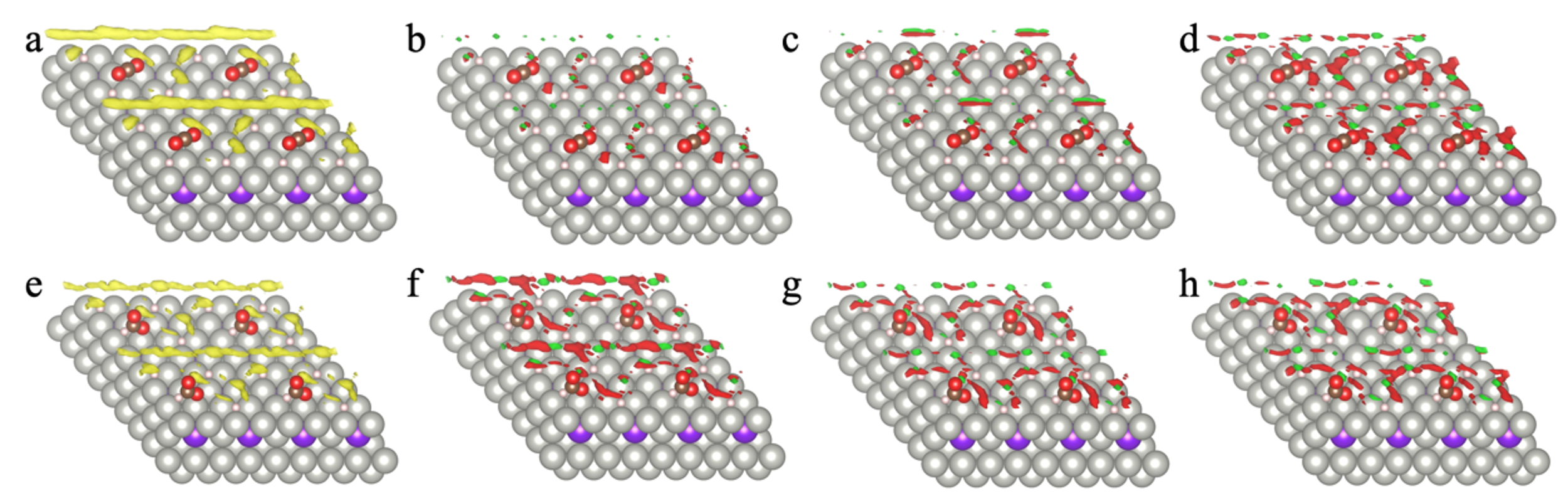


**Figure S5**. Three-dimensional (3D) density maps for CO_2_ distribution near the catalyst surface at the transition state of elementary reaction *CO2 + *H → *HCOO at 500 K under varying CO_2_ bulk densities. a. Density map at the initial CO_2_ adsorption configuration with ρ_bulk-CO2_ = 0.00001 molecules/Å^3^. b–d. Differential density maps showing changes as ρ_bulk-CO2_ increases from 0.00001 to 0.0001 (b), 0.0001 to 0.0005 (c), and 0.0005 to 0.003 molecules/ Å^3^ (200 bar) (d) for the initial state. e. Density map at the *HCOO transition state with ρ_bulk-CO2_ = 0.00001 molecules/ Å^3^. f–h. Differential density maps for the *HCOO transition state as ρ_bulk-CO2_ increases from 0.00001 to 0.0001 (f), 0.0001 to 0.0005 (g), and 0.0005 to 0.003 molecules/ Å^3^ (200 bar) (h). All simulations were conducted at 500 K with a fixed hydrogen bulk density of ρ_bulk-H2_ = 0.00001 molecules/ Å^3^. The isosurface value is set at 1×10^-6^ molecules/ Å^3^.


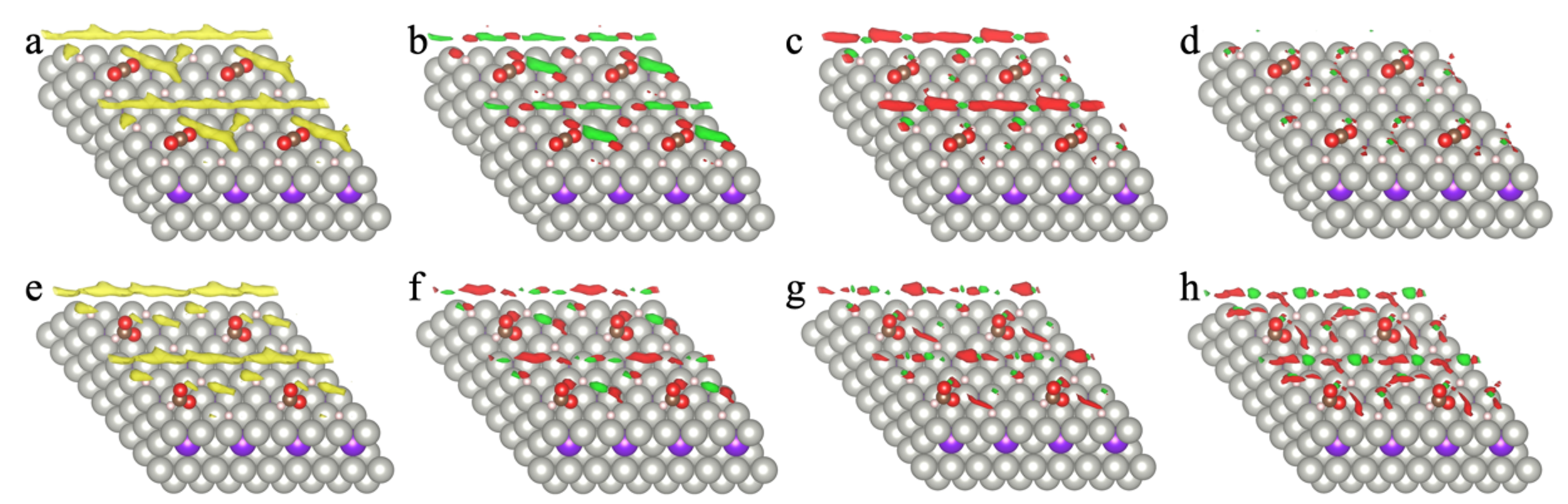


**Figure S6**. Three-dimensional (3D) density maps for CO_2_ distribution near the catalyst surface at the transition state of elementary reaction *CO2 + *H → *HCOO at 700 K under varying CO_2_ bulk densities. a. Density map at the initial CO_2_ adsorption configuration with ρ_bulk-CO2_ = 0.00001 molecules/Å^3^. b–d. Differential density maps showing changes as ρ_bulk-CO2_ increases from 0.00001 to 0.0001 (b), 0.0001 to 0.0005 (c), and 0.0005 to 0.002 molecules/ Å^3^ (200 bar) (d) for the initial state. E. Density map at the *HCOO transition state at ρ_bulk-CO2_ = 0.00001 molecules/ Å^3^. f–h. Differential density maps for the *HCOO transition state as ρ_bulk-CO2_ increases from 0.00001 to 0.0001 (f), 0.0001 to 0.0005 (g), and 0.0005 to 0.002 molecules/ Å^3^ (200 bar) (h). All simulations were conducted at 300 K with a fixed hydrogen bulk density of ρ_bulk-H2_ = 0.00001 molecules/ Å^3^. The isosurface value is set at 1×10^-6^ molecules/ Å^3^.


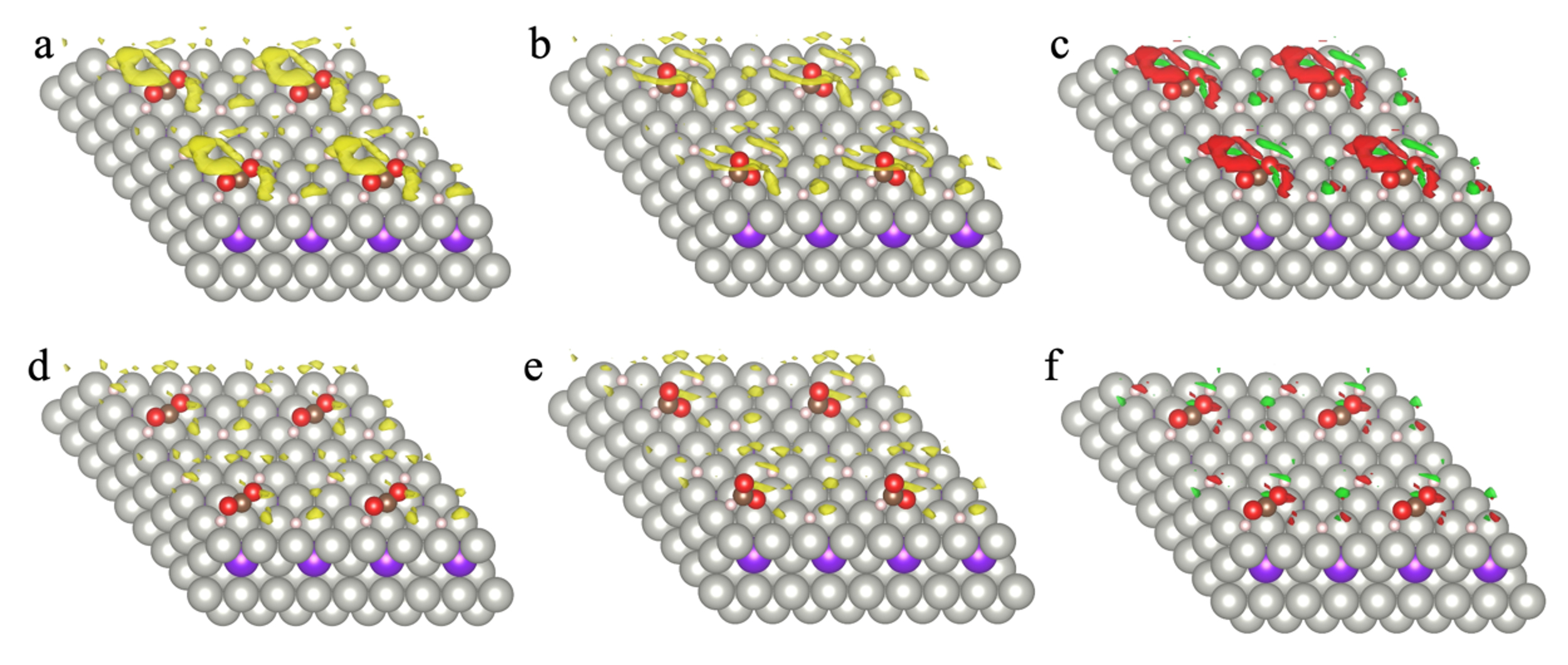


**Figure S7**. The local H_2_ density near the catalyst surface at the transition states of two hydrogenation pathways. a–c. At 300 K with ρ_bulk-CO2_ = 0.00001 molecules/Å^3^ (0.4 bar) and ρ_bulk-H2_ = 0.004 molecules/ Å^3^ (180 bar): a) H_2_ density map around the *COOH transition state. B) H_2_ density map around the *HCOO transition state. c) Differential density map of H_2_ (ρ_TSCOOH_ – ρ_TSHCOO_) between the two transition states. d–f. At 300 K with ρ_bulk-CO2_ = 0.001 molecules/ Å^3^ (35 bar) and ρ_bulk-H2_ = 0.001 molecules/ Å^3^ (42 bar): d. H_2_ density map around the *COOH transition state. e. H_2_ density map around the *HCOO transition state. f. Differential density map of H_2_ between the two transition states. The isosurface value is set at 1×10^-8^ molecules/ Å^3^.


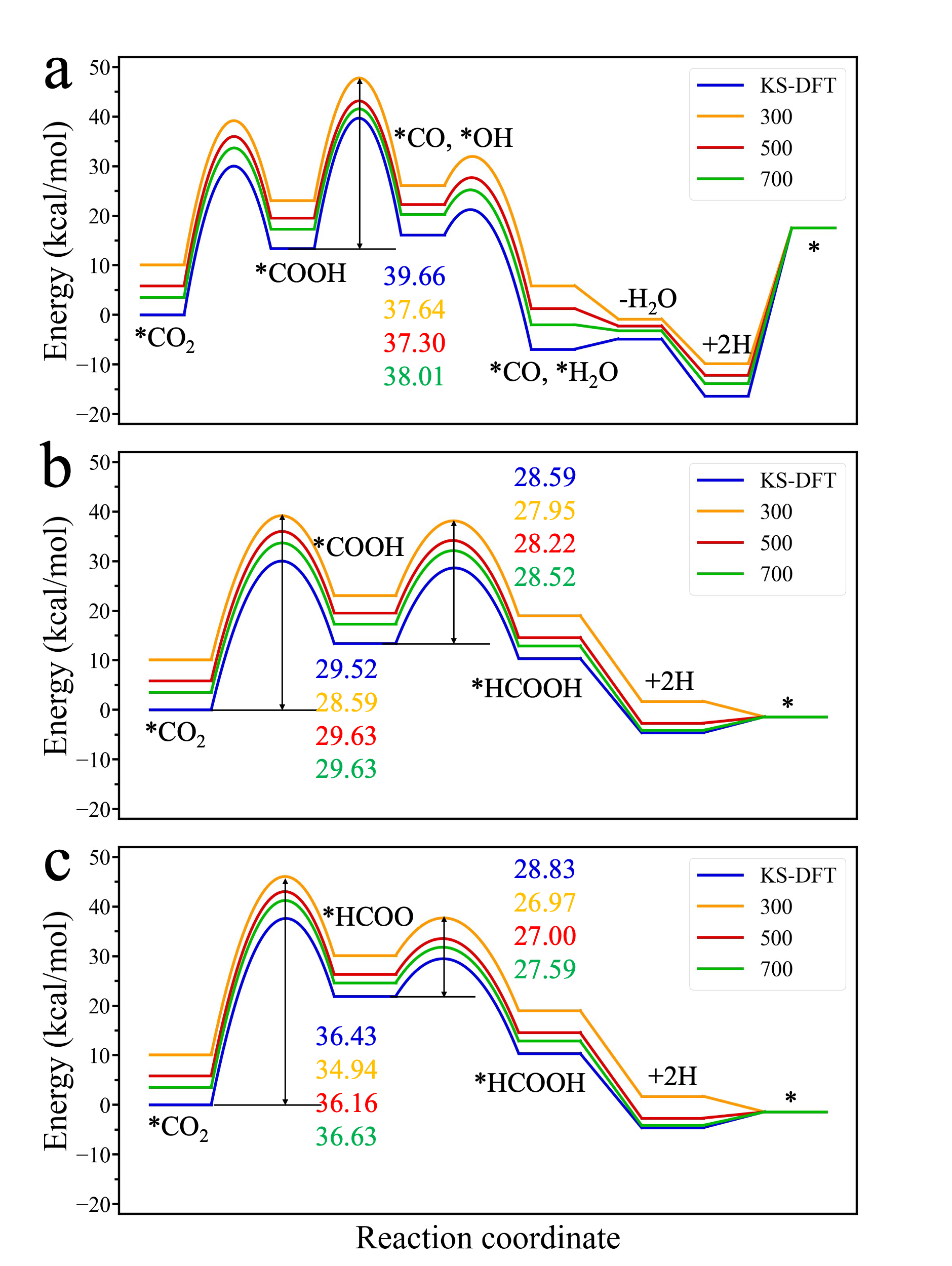


**Figure S8**. Reaction energy profiles for CO_2_ hydrogenation on the PdZn_H075 surface. a. CO formation via CO_2_ hydrogenation. b. HCOOH formation via the *COOH pathway. c. HCOOH formation via the *HCOO pathway. Grand potential profiles were calculated at 300 K, 500 K, and 700 K under gas-phase conditions of P_CO2_ = 10 bar and P_H2_ = 30 bar.


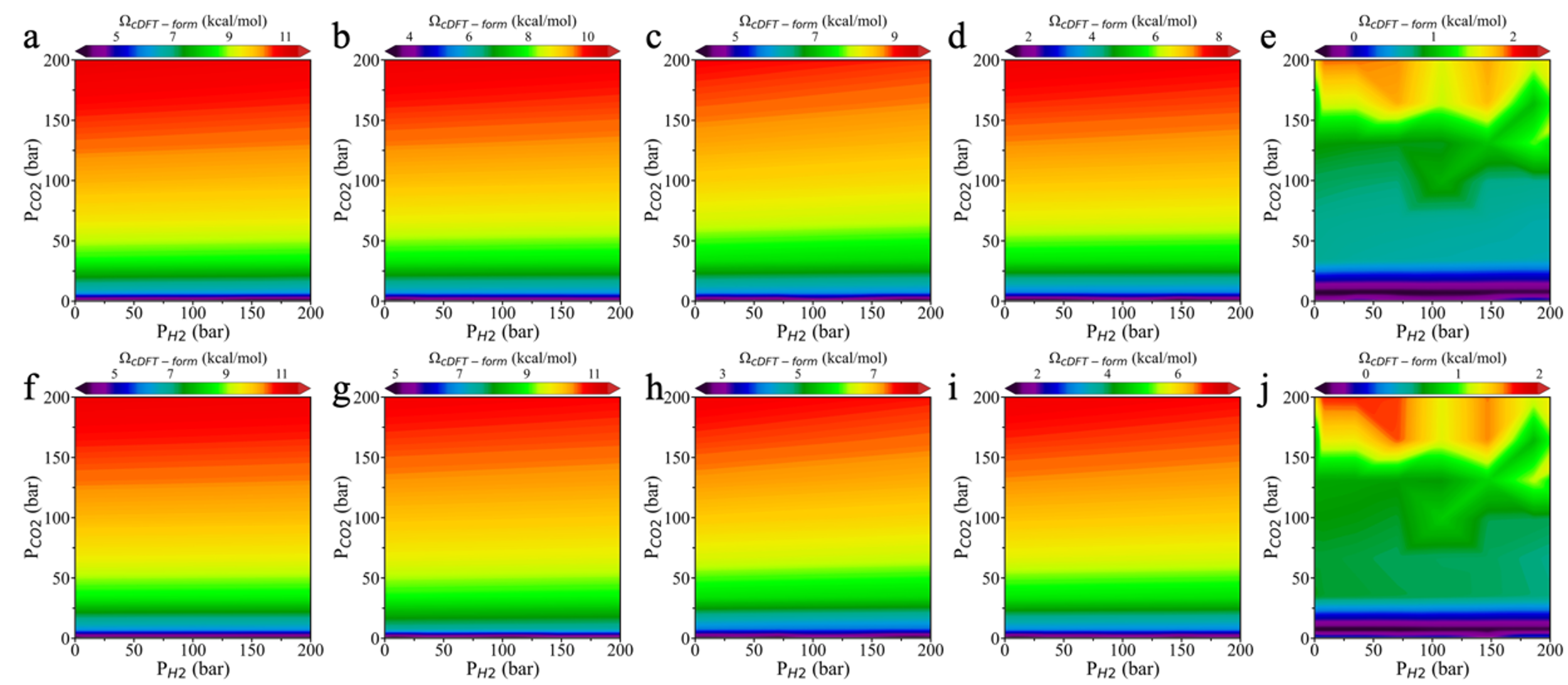


**Figure S9**. Grand-potential corrections of the formation energy (Ω_cDFT-form_) for *COOH and *HCOO intermediates as a function of gas-phase partial pressures at 500 K. a–e, Ω_cDFT-form_ for the *COOH intermediate on PdZn_H000 (a), PdZn_H025 (b), PdZn_H075 (c), PdZn_H100 (d), and PdZn_H075_CO025 (e). f–j. Ω_cDFT-form_ for the *HCOO intermediate on the corresponding surfaces.


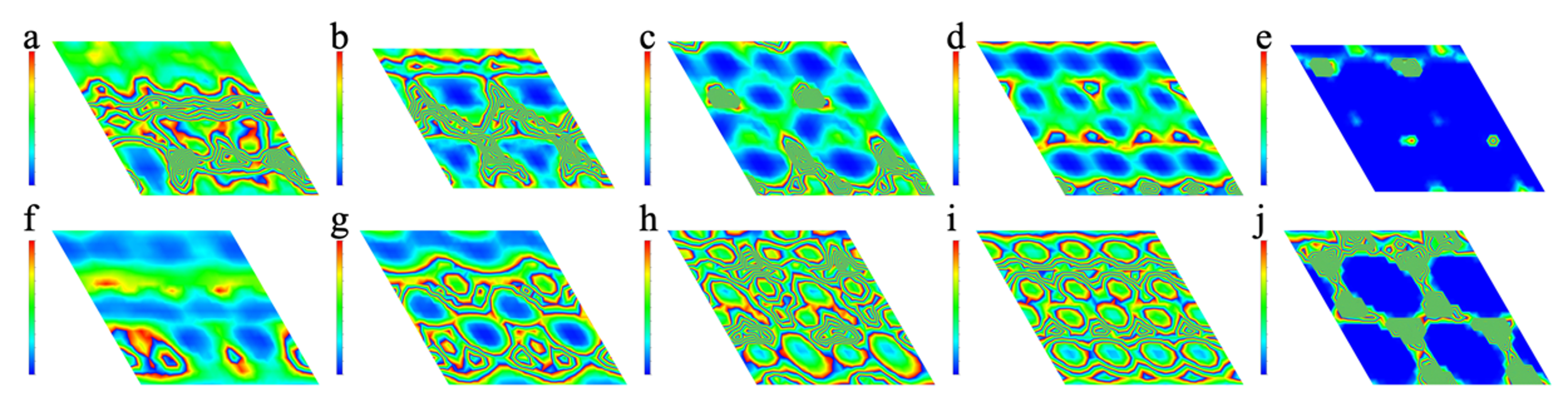


**Figure S10**. Two-dimensional gas-density maps at 2.5 Å above the surface under various surface coverages. a–e. CO_2_ density maps for surfaces with one surface H atom (a), PdZn_H025 (b), PdZn_H075 (c), PdZn_H100 (d), and PdZn_H075_CO025 (e). f–j. Corresponding H_2_ density maps for the same surfaces. All simulations were performed at 500 K with P_CO2_ = 10 bar and P_H2_ = 30 bar. The isosurface value is 1×10^-6^ molecules/Å^3^ for CO_2_ and 1×10^-8^ molecules/Å^3^ for H_2_.


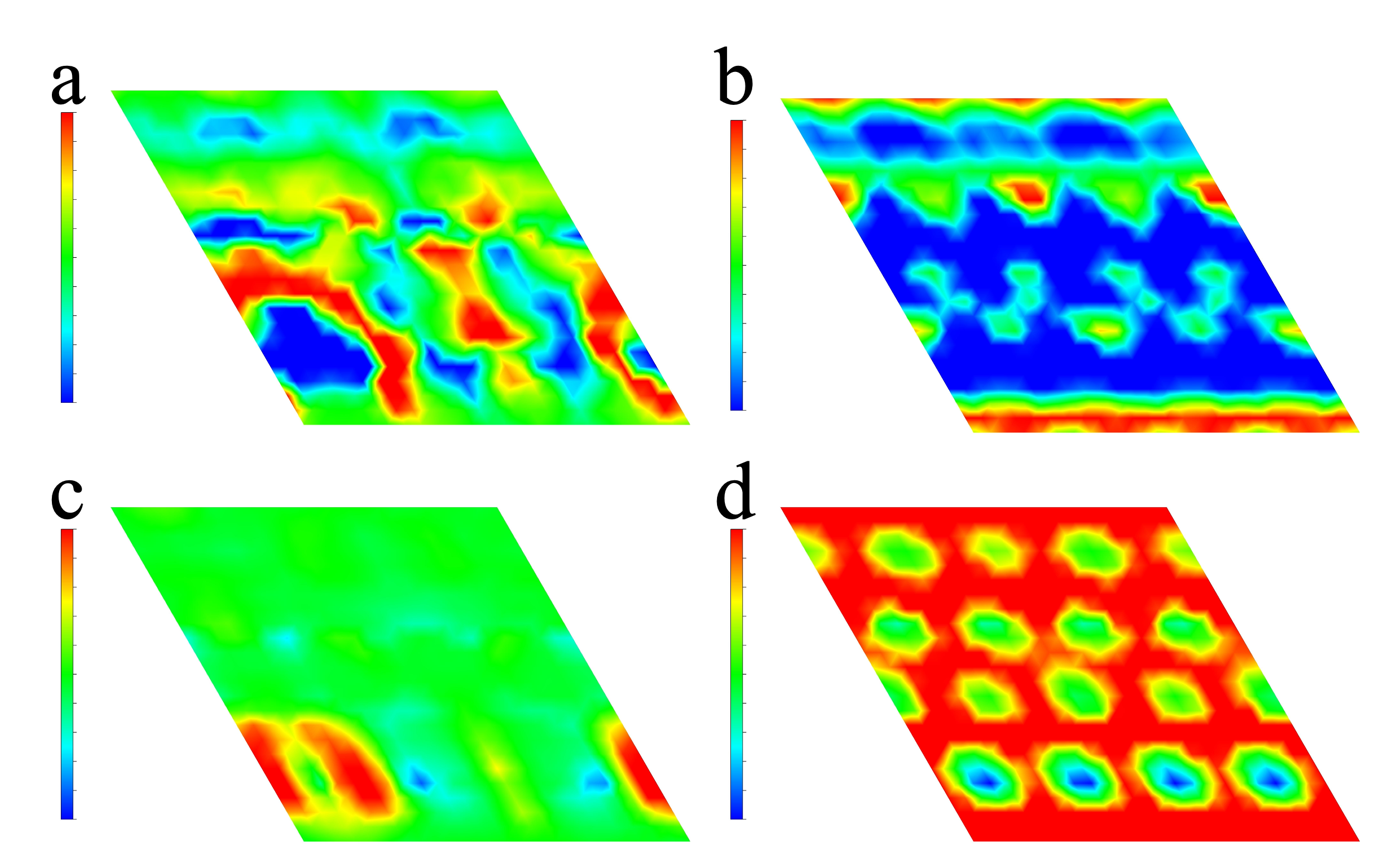


**Figure S11**. Two-dimensional differential density maps at 2.5 Å above the catalyst surface. a–b. CO_2_ differential density maps comparing a surface with one adsorbed H atom (a) and PdZn_H100 (b) to the PdZn_H000 surface. c–d. Corresponding H_2_ differential density maps. All simulations were performed at 500 K with P_CO2_ = 10 bar and P_H2_ = 30 bar. The isosurface value is 1×10^-6^ molecules/Å^3^ for CO_2_ and 1×10^-8^ molecules/Å^3^ for H_2_.


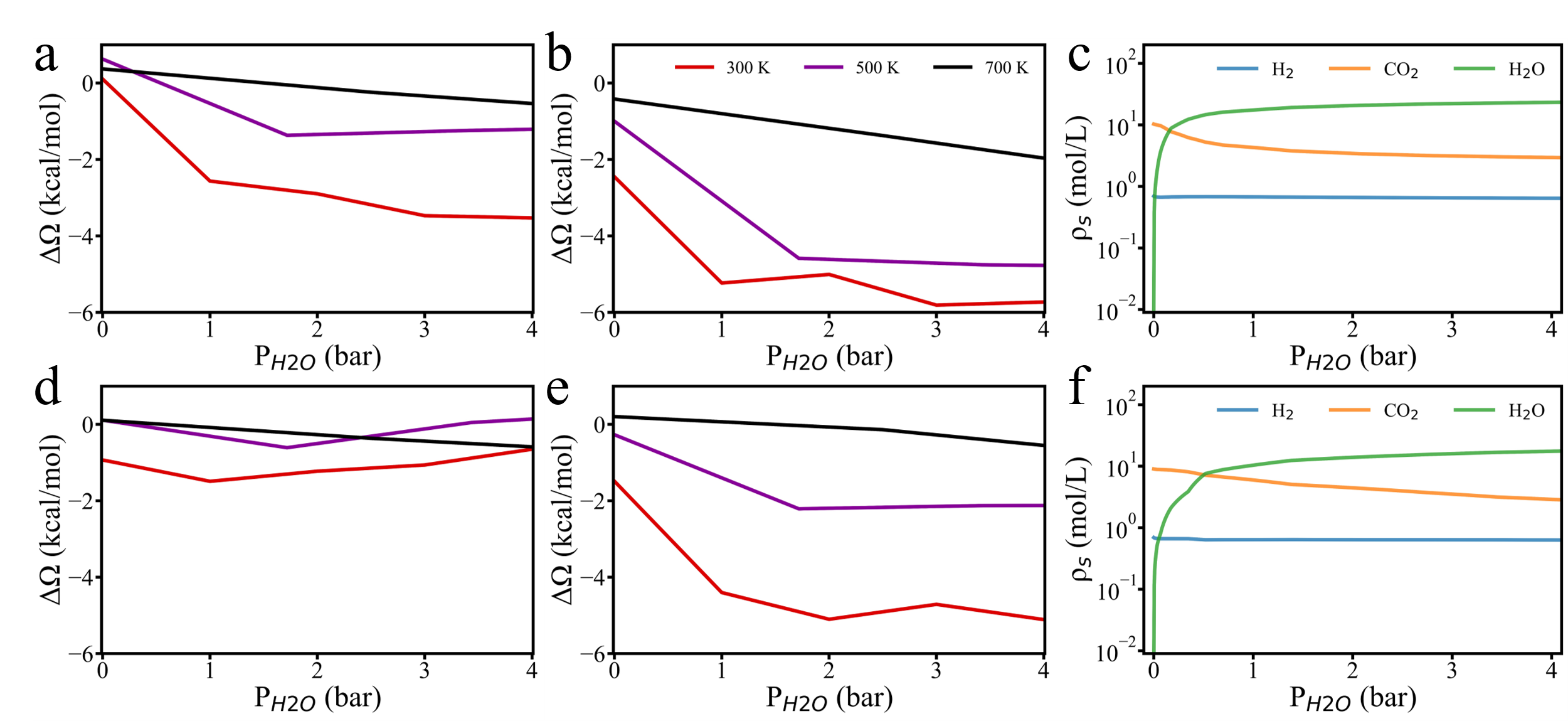


**Figure S12**. The influence of H_2_O partial pressure on CO_2_ hydrogenation. Variation of the grand potential barrier for CO_2_ hydrogenation to *COOH on PdZn_H025 (a) and to *HCOO (b). (c) Surface gas densities on PdZn_H025 *CO_2_ intermediates under 500 K. Variation of the grand potential barrier for CO_2_ hydrogenation to *COOH on PdZn_H075 (a) and to *HCOO (b). (c) Surface gas densities on PdZn_H075 *CO_2_ intermediates under 500 K. All simulations were conducted under the conditions of bulk gas-phase partial pressures P_CO2_ = 10 bar and P_H2_ = 30 bar.

**
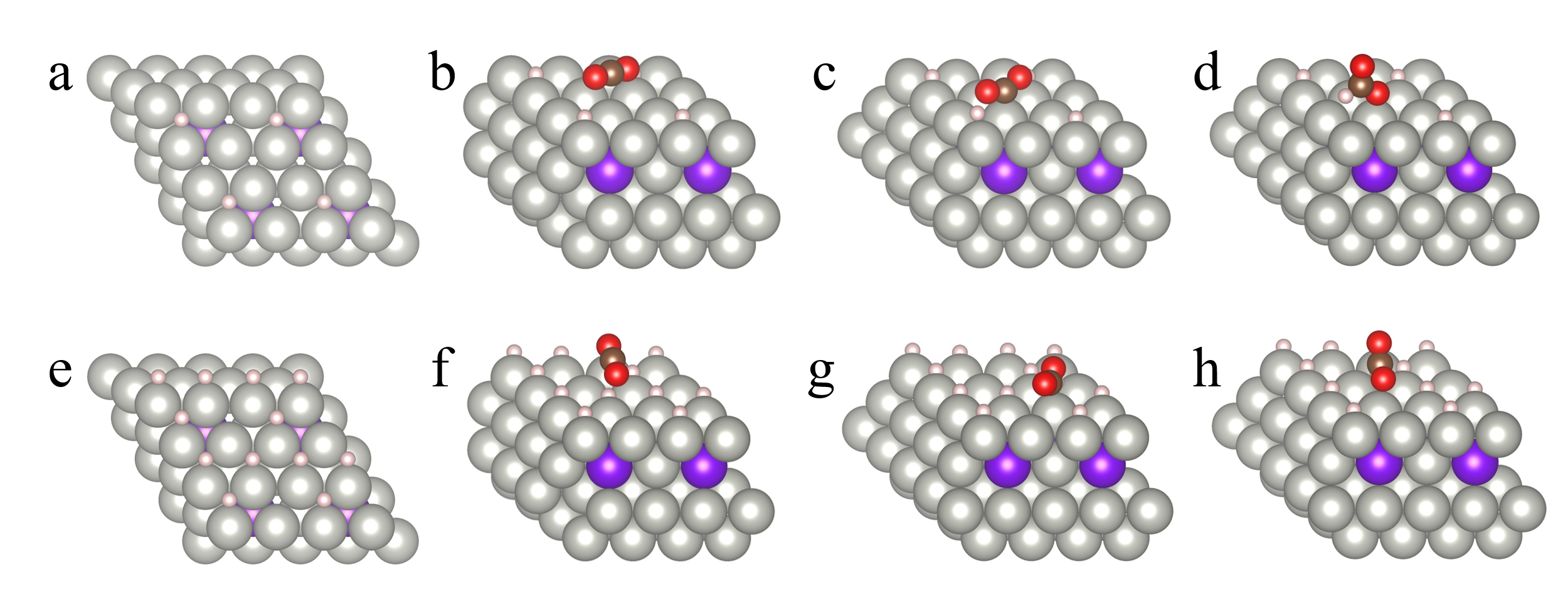
**

**Figure S13**. Structure of the PdZn surface with various key intermediates. a. PdZn surface with 0.25 H coverage (PdZn_H025), showing CO_2_ adsorption (b), *COOH transition state (c), and *HCOO transition state (d). e. PdZn surface with 0.75 H coverage (PdZn_H075), along with the CO_2_ adsorption (f), *COOH transition state (g), and HCOO transition state (h) intermediates. The silver, purple, brown, red, and pinkish-white spheres represent Pd, Zn, C, O, and H atoms, respectively. The atomic configurations of all intermediates were obtained from Zhang and Liu.^[6,7]^

**References:**

[1] B. Peters, Chapter 10 - Transition state theory. in *Reaction Rate Theory and Rare Events Simulations* (Ed.: B. Peters), Elsevier, Amsterdam **2017**, pp. 227–271.

[2] M. Zhou, J. Wu, A GPU implementation of classical density functional theory for rapid prediction of gas adsorption in nanoporous materials. *J. Chem. Phys.* **2020**, *153*, 074101.

[3] F. M. Mourits, F. H. A. Rummens, A critical evaluation of Lennard–Jones and Stockmayer potential parameters and of some correlation methods. *Can. J. Chem.* **1977**, *55*, 3007.

[4] S. Zhen, G. J. Davies, Calculation of the Lennard-Jonesn–m potential energy parameters for metals. *Phys. Status Solidi A* **1983**, *78*, 595.

[5] A. K. Rappe, C. J. Casewit, K. S. Colwell, W. A. Goddard, W. M. Skiff, UFF, a full periodic table force field for molecular mechanics and molecular dynamics simulations. *J. Am. Chem. Soc.* **1992**, *114*, 10024.

[6] H. Zhang, P. Liu, Fine-tuning catalytic selectivity by modulating catalyst-environment interactions: CO2 hydrogenation over Pd-based catalysts. *Chem Catal.* **2025**, *5*, 101156.

[7] H. Zhang, X. Wang, P. Liu, Reaction-driven selective CO2 hydrogenation to formic acid on Pd(111). *Phys. Chem. Chem. Phys.* **2022**, *24*, 16997.
